# Self-serving reward and punishment: evidence from the laboratory

**DOI:** 10.1038/s41598-023-41256-5

**Published:** 2023-08-26

**Authors:** Jianing Chen, Zeng Lian, Jie Zheng

**Affiliations:** 1 International Business School, Beijing Foreign Studies University, Beijing, 100089 China; 2 Center for Economic Research, Shandong University, Jinan, 250100 China

**Keywords:** Psychology, Human behaviour

## Abstract

Reward for altruism and punishment for selfishness are crucial components for the maintenance of society. Past studies have provided strong evidence that people are willing to incur costs to punish selfish behaviors and to reward altruistic behaviors, but how their willingness to do so depends on their relationship with the individuals conducting the anti-social or pro-social behaviors is much less explored. To probe into this question, we devised a three-stage experiment that combined a revised dictator game and third-party reward or punishment. We employed two payoff frameworks, alignment and conflict, and analyzed how third-party’s willingness to reward and punish differed when their interests were either aligned or in conflict with the first-party under observation. We found that due to considerations for personal interests, third-party’s reward and punishment levels deviated from what was deemed “legitimate” by society, that is, the level of reward and punishment that enhances society’s intrinsic motivations to comply with social norms and act pro-socially. When an anti-social behavior was observed, third-party punished less severely under the alignment framework than under the conflict framework; when a pro-social behavior was observed, third-party demonstrated self-serving reward under the alignment framework, but they rewarded altruistically under the conflict framework. These findings provided evidence for third-party’s self-serving reward and punishment.

## Introduction

In the past several decades, there has been substantial progress in understanding the evolution of pro-social behaviors based on punishment^1–5^. As one important social norm driving pro-social behaviors as well as legitimate sanctioning, altruism has been widely studied in third-party reward and punishment games. In a typical third-party game, first-party conducts a behavior on second-party, either pro-social or anti-social, and this behavior is observed by a disinterested third-party. The existing literature on third-party shows three ways they may react: when the behavior is anti-social, they could either punish perpetrators or compensate victims^6–11^; when the behavior is pro-social, they could reward the altruistic first-party^12–16^. Most studies explore third-party’s preference between different reactive methods and the impact that their response exerts on first-party’s behaviors. However, relatively few studies research into whether third-party will act the same when they no longer stay disinterested. That is, when observers’ own interests are influenced by their third-party decisions, will the frequency and intensity of reward and punishment differ? Moreover, does the answer to this question vary with third-party’s relationship with the first-party? In this paper, we aim to investigate whether and to what extent third-party’s self-serving belief influences their decisions, and how third-party’s relationship with the first-party affects their willingness to reward and punish.

To answer these questions, we better start by understanding the motives behind third-party reward and punishment. When a norm violation is observed, it provokes anger, or moral outrage^17^, and the punishment inflicted by the angered observers is critical in rule enforcement^18^. Seip et al.^19^ finds empirical evidence that angered observers are willing to incur costs to punish. Third-party may also choose to punish in consideration of reputational benefit as they desire to be respected, trusted and valued positively^20–22^. Therefore, punishment for norm violation also becomes a social obligation that third-party feel obliged to carry out by ethical norms and social anticipation^23^. However, when alternatives like compensating victims or withholding help to perpetrators are available, third-party are less likely to punish^3–6^, indicating that punishment is perceived as a weaker signal for trustworthiness and cooperation^21,22,24^. When a pro-social behavior is observed, similar to third-party punishment, observers reward others due to reputational benefit and to ultimately benefit themselves^25^. However, third-party are not always moral. In some cases, they commit second-order free-riding, or they give out reward and punishment that promote anti-social behaviors^26–28^.

In this study, we focus on third-party’s self-serving behaviors. Past studies have long revealed the underlying contradiction in third-party behaviors, which involves a tension between giving legitimate reward and punishment and acting to one’s self-interest^29–31^. Legitimacy, according to its definition from social psychology, is a “judgement by group members that they ought to voluntarily obey social rules irrespective of the likelihood of reward and punishment”^32^. In other words, legitimacy obtains when the features of authoritative institutions, or choices of observing third-party, enhance the intrinsic motivations of the public to carry out social norms and act pro-socially^33^. When third-party give out reward and punishment legitimately, they act in accordance with society’s anticipation of third-party behaviors, enhance society’s intrinsic motivations to behave pro-socially, and gain a positive self-image in this process^20,21,25^. However, when their own interests are influenced by their third-party decisions, they may deviate from social anticipation for legitimate third-party behaviors in order to obtain more personal interests when they can find excuses for themselves^23^. This indicates that third-party care more about maintaining a positive social image rather than actual adherence to moral principles^34,35^, and they tend to gain more personal interests without explicitly revealing their true self-serving preference.

Another key focus of this study is the interest relationship between first-party and third-party, which is denoted by third-party’s payoff scheme, and we aim to explore how interest relationship influences third-party behaviors. Past studies have revealed several incentives that influence third-party behaviors: intrinsic motives include third-party’s perception of social norms such as altruism, and their moral willingness to comply with them; extrinsic motives include payoff schemes and decision transparency^33,36,37^. Among all the factors, Alventosa et al.^37^ finds solid evidence that payoff schemes of third-party are the driver of experimental outcomes and exert a huge influence not only on third-party behaviors, but also on the behaviors of the observed individuals. In third-party punishment game, existing literature shows two types of interest relationships: third-party’ interests are either diminished or boosted by their punishment decisions. Correspondingly, these two interest relationships lead to two types of third-party punishment: costly punishment and profit-seeking punishment. Costly punishment is inflicted when third-party sacrifice personal interest to punish transgressors. Kriss et al.^23^ point out that, although third-party exhibit a tendency to incur costly punishment, they are secretly reluctant and incline to circumvent the responsibility to punish when they can do so without explicitly revealing that this is their true preference. Similarly, fewer instances of costly punishment are observed when other forms of responses are available, such as costly compensation^11^ or withholding help to perpetrators^13,38^. In contrast to costly punishment, the other type of punishment, profit-seeking punishment enables third-party to gain profit by depriving violators of resources and capturing these resources themselves. According to^39^, third-party punish more harshly in profitable treatments than in non-profitable ones, and profit-seeking punishment substantially degrades punishment’s efficacy of encouraging norm conformity. Though these two types of punishment are both analyzed, scarcely any studies combine them and examine how third-party punishment varies when observers’ personal interest is either harmed or boosted by the punishment behavior, leaving room for further study into third-party’s self-serving punishment.

Similar to punishment, in third-party reward game, previous studies find that when third-party have to reward at their own expense, the demand for costly reward is substantially lower if they can also withhold punishment as a form of costless reward^13^. In^16^, Jana and Daan delve into different types of costly reward and conclude that net positive reward, where the benefit of receiving a reward is higher than the cost of providing it, is more effective in sustaining cooperation than transfer reward. This indicates that when the interests of third-party and first-party are in conflict and third-party have to sacrifice personal interests to reward, they are usually reluctant to do so, revealing their self-serving reward. Although third-party reward is not as thoroughly investigated as punishment, earlier results suggest reward to be less efficient than punishment in terms of promoting social norms^12,14,15^.

While third-party reward and punishment are of high research interest, they are mostly studied separately from one another. In addition, “costly” and “profit-seeking” reward and punishment, namely, the interest relationships between first-party and third-party, also lack overall analysis. Therefore, this study extends prior research by applying two unified frameworks that combine reward and punishment with “costly” and “profit-seeking” interest relationships. Observers make third-party decisions under either alignment or conflict framework: under the alignment framework, the interests of first-party and third-party are positively correlated, that is, both win or both lose; whereas under the conflict framework, the interests of first-party and third-party are conflicting, that is, one gains at the cost of the other. Third-party can reward, punish or stay indifferent to pro-social or anti-social behaviors, and their decisions are largely dependent upon their interest relationship with the first-party.

Our experimental results provide solid evidence for third-party’s self-serving reward and punishment. When an anti-social behavior is observed, due to consideration for personal interests, third-party punish lightly when their interests align with first-party, showing their reluctance for “costly” punishment; in contrast, they punish harshly when interests are conflicting, demonstrating “profit-seeking” punishment. When a pro-social behavior is observed, third-party reward generously under the alignment framework, providing evidence for self-serving reward; however, under the conflict framework, contrary to our expectation of third-party rewarding reluctantly, they reward altruistically at the expense of their own interests.

## Experimental design

### The experiment

We devise a three-stage experiment that combines a revised small-stake dictator game with a large-stake third-party reward and punishment game. First, subjects are randomly divided into three-people groups and are assigned the role of Player I, II or III. Each role follows a set of instructions strictly without knowing the experimental content of the other two roles.

**STAGE 1**—The first stage is a small-stake dictator game. Player I is asked to decide the value of X (0 or 1), which determines the payoffs of Player I and II in stage 1. Player I needs to make this allocation decision under one of the two payoff frameworks, either alignment or conflict. Under the alignment framework, the interests of Player I and Player II are positively correlated; under the conflict framework, Player I and Player II have conflicting interests. Specific calculation formulas of Player I and II’s payoffs (P$_I$ and P$_{II}$) in stage 1 under each payoff framework are as follows:

$$\begin{aligned} \begin{aligned} {\textit{Alignment}}:&\; P_I=2+X; P_{II}=3X\\ {\textit{Conflict}}:&P_I=2+X; P_{II}=3-3X \end{aligned} \end{aligned}$$

**STAGE 2**—The second stage is a large-stake third-party reward and punishment game. First, we inform Player III of Player I’s decision in the first stage. Then, Player III is required to decide the value of Y (integers from 0 to 10), which determines the payoffs of Player III and I in Stage 2. Player III’s decision is also put under one of the two payoff frameworks: under the alignment framework, the payoffs of Player III and Player I are positively correlated; whereas under the conflict framework, Player III and Player I have conflicting interests. Specific calculation formulas of Player III and I’s payoffs ($\hbox {P}_{III}$ and $\hbox {P}_I$) in stage 2 under each payoff framework are listed below:

$$\begin{aligned} \begin{aligned} {\textit{Alignment}}:&\; P_{III}=20+Y; P_I=3Y\\ {\textit{Conflict}}:&P_{III}=20+Y; P_I=30-3Y \end{aligned} \end{aligned}$$

**STAGE 3**—The third stage is an estimation stage and we provide incentives for all the estimations. First, Player I needs to estimate Player III’s decision in stage 2 without knowing Player III’s actual decision. The closer the Y-value estimated by Player I is to the actual Y-value decided by Player III, the more token will be rewarded. The incentive on this estimation is calculated as follows:

Assuming that Player I’s estimation of Y-value is G and Player III’s actual decision of Y-value is T, Player I receives a $\hbox {bonus} = 10*[1-((T-G)/10)^ 2]$. That means, Player I receives the highest bonus when his estimation G equals Player III’s actual decision T.

Next, Player II needs to make two estimations without knowing either Player I’s or Player III’s decisions. Player II needs to first estimate Player I’s decision in stage 1. If the estimation is correct, Player II will be rewarded with 5 tokens, otherwise he receives none. Then, Player II needs to estimate Player III’s decision in stage 2 under two conditions, $\hbox {X}=0$ or $\hbox {X}=1$. The calculation of the incentive on this estimation is similar to the one on Player I’s estimation.

**Figure 1:**
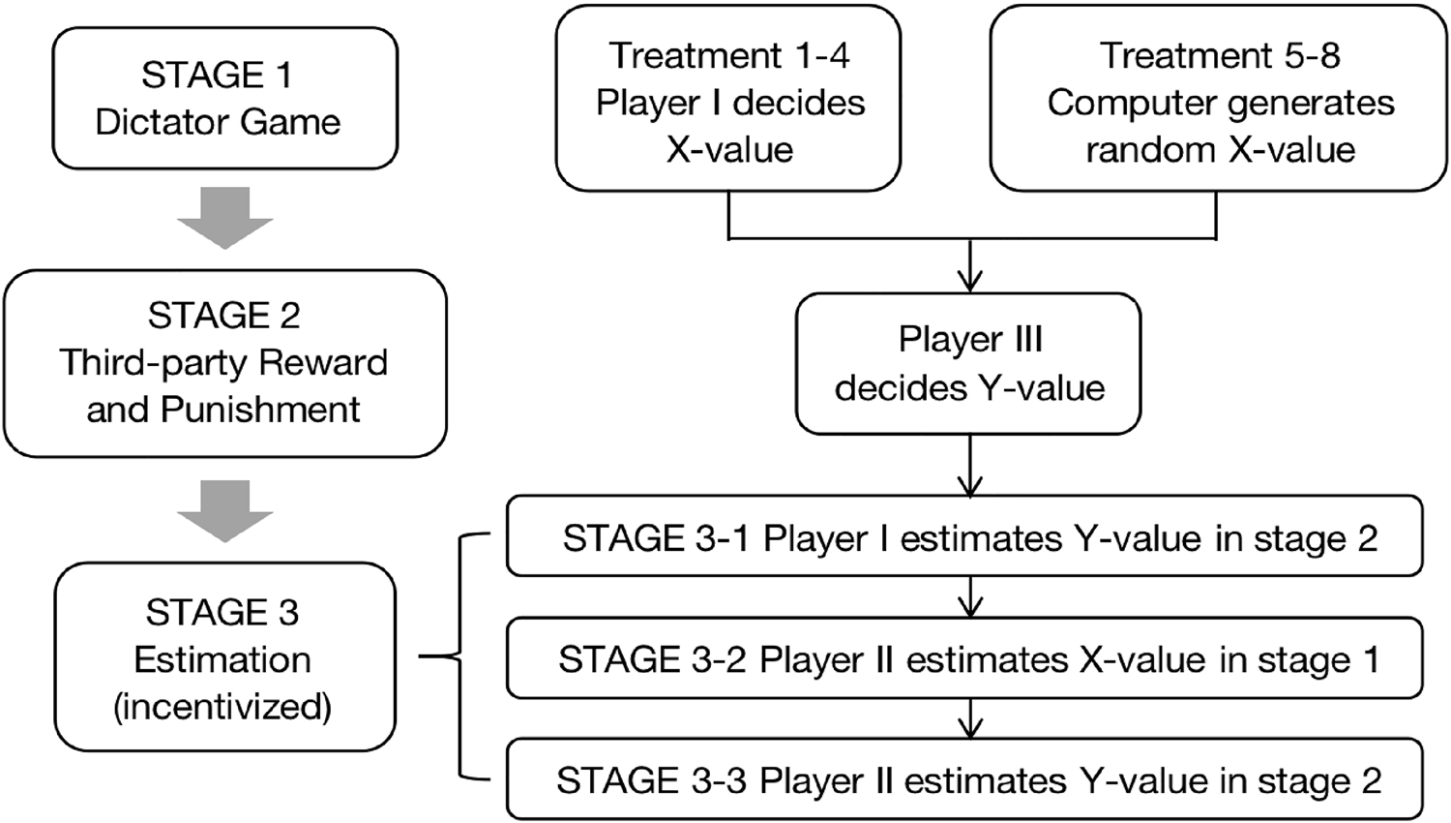
Flow chart of the experimental stages.

By providing incentives, we elicit Player Is’ and Player IIs’ beliefs about the level of third-party reward and punishment. Player IIs’ estimations are relatively objective, and since they remain ignorant of both Player Is’ and Player IIIs’ decisions, their estimations can serve as social anticipation for third-party reward and punishment, that means, Player IIs’ estimations of Player IIIs’ decisions represent the level of third-party reward and punishment that the society considers legitimate to exert on the first-party conducting the pro-social or anti-social behavior. On the contrary, Player Is’ estimations are biased as they themselves are the first party under observation. Below Fig. 1 is the flowchart of the experimental stages.

The three stages form one complete round, and subjects’ payoffs are revealed before the next round starts. Every treatment consists of three rounds in total. Throughout the three rounds, subjects’ role, Player I, II or III, stays the same, so they remain ignorant of the experimental procedure of the other two roles. However, subjects’ group members reshuffle at the beginning of each round, so in each round, subjects have to make decisions in interaction with newly assigned group members and their decisions are not influenced by the results of former rounds. At the end of the entire treatment, the system randomly selects one round, the payoff of which is output as subjects’ final profit.

Table 1 provides detailed information on all our treatments. As both Player I and Player III’s decisions are put under either alignment or conflict framework, we list 4 possible payoff framework combinations and come up with main treatment 1–4. Noticeably, treatment 5–8 are benchmark groups, where X-values in stage 1 are not determined by Player Is but are randomly assigned by computers, and Player IIIs need to decide the value of Y on this basis.

**Table 1:** Treatment.

Treatment	STAGE 1: dictator game	STAGE 2: third-party reward and punishment	STAGE 3: estimation
T1: A–A	Alignment: $\hbox {P}_I=2+\hbox {X}$; $\hbox {P}_{II}=3\hbox {X}$	Alignment: $\hbox {P}_{III}=20+\hbox {Y}$; $\hbox {P}_I=3\hbox {Y}$	Incentivized
T2: A–C	Alignment: $\hbox {P}_I=2+\hbox {X}$; $\hbox {P}_{II}=3\hbox {X}$	Conflict: $\hbox {P}_{III}=20+\hbox {Y}$; $\hbox {P}_I=30-3\hbox {Y}$	Incentivized
T3: C–A	Conflict: $\hbox {P}_I=2+\hbox {X}$; $\hbox {P}_{II}=3-3\hbox {X}$	Alignment: $\hbox {P}_{III}=20+\hbox {Y}$; $\hbox {P}_I=3\hbox {Y}$	Incentivized
T4: C–C	Conflict: $\hbox {P}_I=2+\hbox {X}$; $\hbox {P}_{II}=3-3\hbox {X}$	Conflict: $\hbox {P}_{III}=20+\hbox {Y}$; $\hbox {P}_I=30-3\hbox {Y}$	Incentivized
T5: B–A–A	Alignment (random X): $\hbox {P}_I=2+\hbox {X}$; $\hbox {P}_{II}=3\hbox {X}$	Alignment: $\hbox {P}_{III}=20+\hbox {Y}$; $\hbox {P}_I=3\hbox {Y}$	Incentivized
T6: B–A–C	Alignment (random X): $\hbox {P}_I=2+\hbox {X}$; $\hbox {P}_{II}=3\hbox {X}$	Conflict: $\hbox {P}_{III}=20+\hbox {Y}$; $\hbox {P}_I=30-3\hbox {Y}$	Incentivized
T7: B–C–A	Conflict (random X): $\hbox {P}_I=2+\hbox {X}$; $\hbox {P}_{II}=3-3\hbox {X}$	Alignment: $\hbox {P}_{III}=20+\hbox {Y}$; $\hbox {P}_I=3\hbox {Y}$	Incentivized
T8: B–C–C	Conflict (random X): $\hbox {P}_I=2+\hbox {X}$; $\hbox {P}_{II}=3-3\hbox {X}$	Conflict: $\hbox {P}_{III}=20+\hbox {Y}$; $\hbox {P}_I=30-3\hbox {Y}$	Incentivized

In treatment 1–4, the first capital letter indicates the payoff framework used in stage 1, and the second capital letter indicates the payoff framework used in stage 2. A stands for alignment framework, C stands for conflict framework. In treatment 5–8, there is a capital letter B, which stands for benchmark.

### Hypothesis

We formed three hypotheses before conducting the experiment. All the hypotheses are formally discussed and recorded by the Behavioral and Experimental Social Science Laboratory at Beijing Foreign Studies University (Registration No. 2022H006). The hypotheses are as follows:

#### Third-party reward and punishment

In all the treatments, Player III needs to decide on a Y-value based on Player I’s previous allocation decision. Based on numerous past studies that probe into third-party reward and punishment^6–16^, we predict that third-party tend to reward altruistic first-party generously and be harsh on those who act selfishly, even when reward or punishment is costly. Based on this prediction, we come up with Hypothesis 1a as follows:

##### **Hypothesis 1a**

When the interests of third-party and first-party are positively correlated, third-party will decide on higher Y-values when first-party act pro-socially as opposed to anti-socially. When the interests of third-party and first-party are in conflict, third-party will decide on lower Y-values when first-party act pro-socially as opposed to anti-socially.

However, in benchmark groups, as X-values are randomly assigned by computers, third-party’s decisions do not include reward or punishment factors. In treatment 5 and 7, when the interests of third-party and first-party are positively correlated, third-party will decide on a Y-value that maximize both parties’ gains no matter X-value equals to 0 or 1. In treatment 6 and 8, when the interests of third-party and first-party conflict with one another, third-party will feel empathetic for first-party and be relatively generous at their own expense. Comparing our predictions of the main treatment groups with that of the benchmark groups, we formulate Hypothesis 1b:

##### **Hypothesis 1b**

When the interests of third-party and first-party are positively correlated, and X-value, either decided by Player I or assigned by the computer, is pro-social, third-party will decide on similar or greater Y-values in main treatments than in benchmark groups, maximizing both parties’ interests; however, when X-value is anti-social, Y-values will be higher in benchmark groups. When the interests of third-party and first-party are in conflict, and X-value is pro-social, Y-values will be higher in benchmark groups; when X-value is anti-social, Y-value will be higher in main treatment groups.

#### Self-serving reward and punishment

Past studies have revealed third-party’s tendency to shirk the responsibility of reward and punish when doing so benefits their personal interests. Szolnoki and Perc^28^ explore second-order free-riding, showing third-party’s unwillingness to shoulder the cost of punishment. Kriss et al.^23^ reveals that third-party punish reluctantly: they choose to avoid the opportunity to punish when they can do so without explicitly revealing that this is their preference. On the contrary, in^39^, third-party fully utilize their power to punish in profitable treatments, gaining more payoff during this process. Based on these previous findings, in our study, since third-party’s payoffs are determined by their own reward and punishment behaviors, we expect to find that third-party’s behaviors are no longer “pure” responses to pro-sociality or anti-sociality, but may deviate from social anticipation for “legitimate” third-party actions. They may shirk or fully utilize their power to reward or punish in order to gain more personal interests. Specifically, under the alignment framework, when a pro-social behavior is observed, Player III may choose Y-value that is close to 10, maximizing both parties’ interests; however, when an anti-social behavior is observed, Player III may punish reluctantly or may even avoid the opportunity to punish. Similarly, under the conflict framework, Player III may severely punish Player I’s anti-social behaviors but reluctantly reward pro-social behaviors, or may even avoid the opportunity to reward. Driven by personal interests, third-party may deviate from the level of reward or punishment considered “legitimate” by society. This social anticipation for legitimate reward and punishment can be reflected by Player IIs’ estimations of Y-values as they remain unknown of Player I and III’s decisions throughout the entire experiment and are relatively detached. The discrepancy between Player IIIs’ actual decisions and Player IIs’ estimations sheds light on third-party’s self-serving reward and punishment. We formulate Hypothesis 2 as follows:

##### **Hypothesis 2**

When the interests of third-party and first-party are positively correlated, and first-party act pro-socially, third-party’s decisions will be similar to social anticipation, maximizing both parties’ interests; however, when first-party act anti-socially, Y-values decided by third-party will exceed social anticipation. When the interests of third-party and first-party are in conflict, no matter first-party act pro-socially or anti-socially, Y-values decided by the third-party will be higher than social anticipation.

#### The amplification effect

Stage 1 is a small-stake dictator game. Player I makes a small-stake decision in stage 1, deciding X-value equals 0 or 1. Stage 2 is a large-stake third-party reward or punishment game. According to previous studies, besides third-party’s responsibility to reward altruism and punish selfishness, they also tend to obtain more personal interests in the process of giving reward and punishment^23,39^. Thus, we expect to find that, in stage 2, due to alignment or conflict of interests, Player III may interpret Player I’s small deed and amplify its implication to justify a certain level of reward or punishment, and gain more payoff in this process. As a result, the X-values that Player Is decide in stage 1 differ by only 1, but Player Is’ payoffs in stage 2 may differ by a very large amount.

##### **Hypothesis 3**

A small difference in decisions by first-party in stage 1 may result in a large difference in payoffs in stage 2. Specifically, payoffs received by pro-social first-party when the interests of first-party and third-party align will be significantly higher than that received by anti-social first-party when interests conflict.

Based on the above hypotheses, we make predictions about the data results. There are four different Y-values that are particularly important to us: *(1) Decision III*$_B$. In benchmark groups, X-value is assigned randomly, thus Player III’s decision has nothing to do with third-party reward and punishment. *(2) Decision III.* Player III’s decision in stage 2 is a third-party response to Player I’s previous decision and includes self-serving preference. *(3) Estimation I.* Player I’s estimation of third-party’s response is strongly biased as Player I himself is the first-party being observed. *(4) Estimation II.* Player II’s estimation of third-party’ response is relatively objective as Player II remains uninvolved and ignorant of Player I and III’s previous decisions. Therefore, estimation II can reflect social anticipation for third-party reward and punishment.

Below in Table 2, we compare the four Y-values in different scenarios:

**Table 2:** Expectation of Y-values.

STAGE 2: framework	STAGE 1: behavior	Decision III and Estimation I and Estimation II	Decision III and Decision III$_B$
Alignment: $\hbox {P}_{III}=20+\hbox {Y}$; $\hbox {P}_{I}=3\hbox {Y}$	Pro-social	Estimation I $\ge$ Decision III $\ge$ Estimation II	Decision III $\ge$ Decision III$_B$
	Anti-social	Estimation I > Decision III > Estimation II	Decision III$_B$ > Decision III
Conflict: $\hbox {P}_{III}=20+\hbox {Y}$; $\hbox {P}_{I}=30-3\hbox {Y}$	Pro-social	Decision III > Estimation II > Estimation I	Decision III$_B$ > Decision III
	Anti-social	Decision III > Estimation II > Estimation I	Decision III > Decision III$_B$

Comparison of Decision III, Estimation I, Estimation II and Decision III$_B$ in four different scenarios: interests of Player I and III are aligned, and Player I is either pro-social or anti-social; and interests of Player I and III are in conflict, and Player I is either pro-social or anti-social.

When the interests of Player III and I are aligned, and Player I’ pro-social behavior is observed, we expect to find all four Y-values to be close to 10. On the contrary, when Player I’s anti-social behavior is observed, society calls for punishment, but due to concern for individual interest, Player III may decide on a Y-value larger than social anticipation, showing Player III’s self-serving punishment; and Estimation I may be even larger, revealing Player I’s tendency to shirk punishment. Decision III will be larger in benchmark groups than in main treatment groups as third-party punishment only exists in the latter.

When the interests of Player III and I are in conflict, and Player I is pro-social, Player III may decide on a Y-value larger than social anticipation, rewarding I reluctantly whereas Player I, seeking more reward, may estimate a Y-value lower than social anticipation. Decision III will be lower in the main treatment groups as it involves third-party reward. When Player I is anti-social, society calls for punishment, and Player III may punish even more harshly as he can benefit from punishment, whereas Player I may estimate a lower Y-value to shirk punishment. Decision III will be larger in the main treatment groups.

## Results

Our main results were drawn from the data collected from the first round of each treatment in which subjects had no clue of what was going to happen next, and their decisions were largely intuitive, eliciting their true beliefs. When the experimental procedures were repeated in the second and third round, subjects grew familiar with the experimental procedure and might develop different strategies. We present these secondary results in the “Additional Analysis–The Learning Effect” section in Methods, exploring how the mentality and strategy of subjects change over time.

### Allocation decisions under small-stake scenario

We first looked into Player Is’ decisions in stage 1. When Player I and II were put under the alignment framework, 91.2% chose to act pro-socially and reach a win–win. Still, 8.8% of participants behaved irrationally and reduced both their and the opponent’s payoff. We believed this phenomenon was caused by free-riding aversion^41^. Since only Player I made decisions in stage 1 but Player II could benefit from this decision, Player I believed that Player II was free-riding. Thus, they would rather sacrifice a little bit of personal payoff to discourage free-riding.

When Player I and II were put under the conflict framework, 66.1% conducted pro-social behaviors while 33.9% acted anti-socially. This indicates that when interests conflict, the majority chose to sacrifice a little of personal gain to benefit others, yet a significant portion deprived their counterparts of any profit to maximize personal gain. It is worth noting here that our design of the conflict framework is a revised version of the standard dictator game used in numerous studies, in which a dictator divides a pie of $10 between himself and a recipient. According to^42^, in the standard dictator game, dictators on average gave 28.35% of the pie, and 36.11% of dictators gave nothing to recipients. Comparing the data, we can find that in our study, the proportion of anti-social Player I who gave nothing to Player II is close to the proportion of dictators who shared nothing in the standard dictator game.

### Third-party reward and punishment

**Figure 2:**
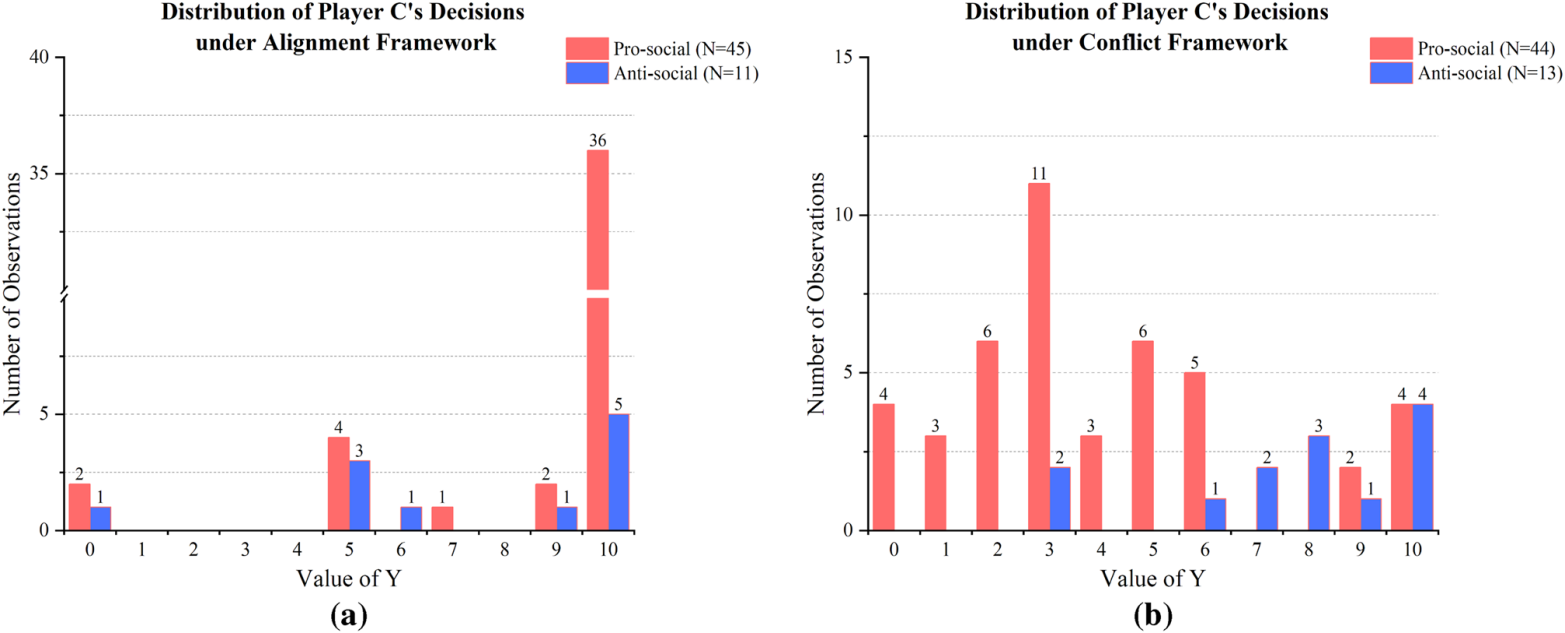
Distribution of Decision III. (**a**) Distribution of Decision III under Alignment Framework ($\hbox {N} = 56$, 45 of Decision IIIs were made when Player I was pro-social, and 11 were made when Player I was anti-social). Decision IIIs were significantly higher when facing pro-social Player I than facing anti-social Player I (Mann–Whitney $\hbox {U}=162.0$, $p=.023$). (**b**) Distribution of Decision III under Conflict Framework ($\hbox {N}=57$, 44 of Decision IIIs were made when Player I was pro-social, and 13 was made when Player I was anti-social). Decision IIIs were significantly lower when facing pro-social Player I than facing anti-social Player I (Mann–Whitney $\hbox {U}=102.5$, $p<.001$). These results reveal third-party reward and punishment.

Our primary interest is in Player III’s rewarding or punitive behaviors under alignment or conflict payoff framework. Below Fig. 2 shows the distribution of Decision III under each payoff framework. In Fig. 2a, the payoffs of Player III and I were aligned, and the Y-values determined by Player III were significantly higher when Player I acted pro-socially than when Player I acted anti-socially (Mann–Whitney $\hbox {U}=162.0$, $p=.023$). This result indicates that in the face of Player I’s altruistic or selfish deed, Player III rewarded altruism and punished selfishness. Yet it is worth noting that, when Player I’s anti-social behavior was observed, nearly half of Player III avoided the opportunity to punish to maximize personal gain, providing evidence for third-party’s self-serving behaviors.

In Fig. 2b, the payoffs of Player III and I were in conflict, and the Y-values determined by Player III were significantly lower when Player I acted pro-socially than when Player I acted anti-socially (Mann–Whitney $\hbox {U}=102.5$, $p<.001$). This result reveals that when Player I was pro-social, Player III decided on lower Y-values, rewarding Player I’s altruism at his own expense, yet some Player III intentionally withheld reward to gain more personal interest. When Player I was anti-social, however, Player III decided on significantly higher Y-values, punishing Player I’s selfishness and meanwhile benefiting from punishment.

These experimental results are consistent with Hypothesis 1a.

#### *Result 1a*

Our findings verify Hypothesis 1a. Under the alignment framework, Y-values decided by third-party are significantly higher when facing pro-social behaviors than anti-social behaviors; under the conflict framework, Y-values decided by third-party are significantly lower when facing pro-social behaviors than anti-social behaviors, providing evidence for third-party reward and punishment.

To further probe into third-party reward and punishment, we conducted Mann–Whitney U Test on Decision III in benchmark groups and main treatment groups. In benchmark groups, as X-values are randomly assigned, Decision III does not involve third-party reward or punishment. By comparing Decision III in main treatment groups and benchmark groups, we aim to reveal third-party reward and punishment. We only found a significant difference when Player III and I were under conflict framework and Player I was pro-social (Mann–Whitney $\hbox {U}=58.5$, $p<.001$). Decision III was smaller in main treatment groups than in benchmark groups, indicating that Player III sacrificed personal interest to reward Player I’s pro-social behavior, providing evidence for third-party reward.

However, no significant difference between punitive Decision IIIs in main treatment groups and in benchmark groups could be discerned. This can probably be explained by^3–6,16,17,19^ that third-party seldom resort to punishment when other options are available since punishment gives out negative signals for cooperation. On the contrary, people are much more willing to give reward to strengthen relationships and foster future cooperation. Thus, Player III’s tendency to reward is stronger and more obvious than punishment.

Based on these findings, we can draw the following conclusion:

#### *Result 1b*

The experimental results are partially consistent with Hypothesis 1b. Under the conflict framework and X-value is pro-social, Y-values are significantly higher in benchmark groups than in main treatment groups, providing evidence for third-party reward. However, there is no significant difference between Y-values in main treatment groups and in benchmark groups in the face of anti-social X-value.

### Self-serving reward and punishment

In this section, we investigate third-party’s self-serving reward and punishment. We have noticed in Fig. 2 that although Player III exhibits the tendency for third-party reward and punishment, however, cases do exist of Player III shirking responsibility of giving reward or punishment when it diminishes their own payoffs. For example, when the payoffs of Player III and I were aligned, half of Player III “neglected” Player I’s selfish deed; when payoffs of Player III and I were in conflict, one-third of Player III turned a blind eye to I’s pro-sociality and left them with no payoffs at all. These results provide evidence for third-party’s self-serving reward and punishment. Third-party tend to avoid the responsibility to reward and punish when rewarding altruism and punishing selfishness harm their own interests.

To further analyze Player III’s self-serving reward and punishment, we compared Decision III with Estimation II. As stated before, Player II is not involved in decision-making and remains ignorant of Player I and III’s decisions, thus Estimation II is relatively objective and reflects the level of third-party reward or punishment that the society deems “legitimate”. However, since under either alignment or conflict framework, Player IIIs’ payoffs are related to their third-party decisions, their reward and punishment decisions are bound to deviate from social anticipation. Thus, by comparing Decision III and Estimation II and revealing how Decision III deviates from Estimation II can we find evidence for Player III’s self-serving reward and punishment.

**Figure 3:**
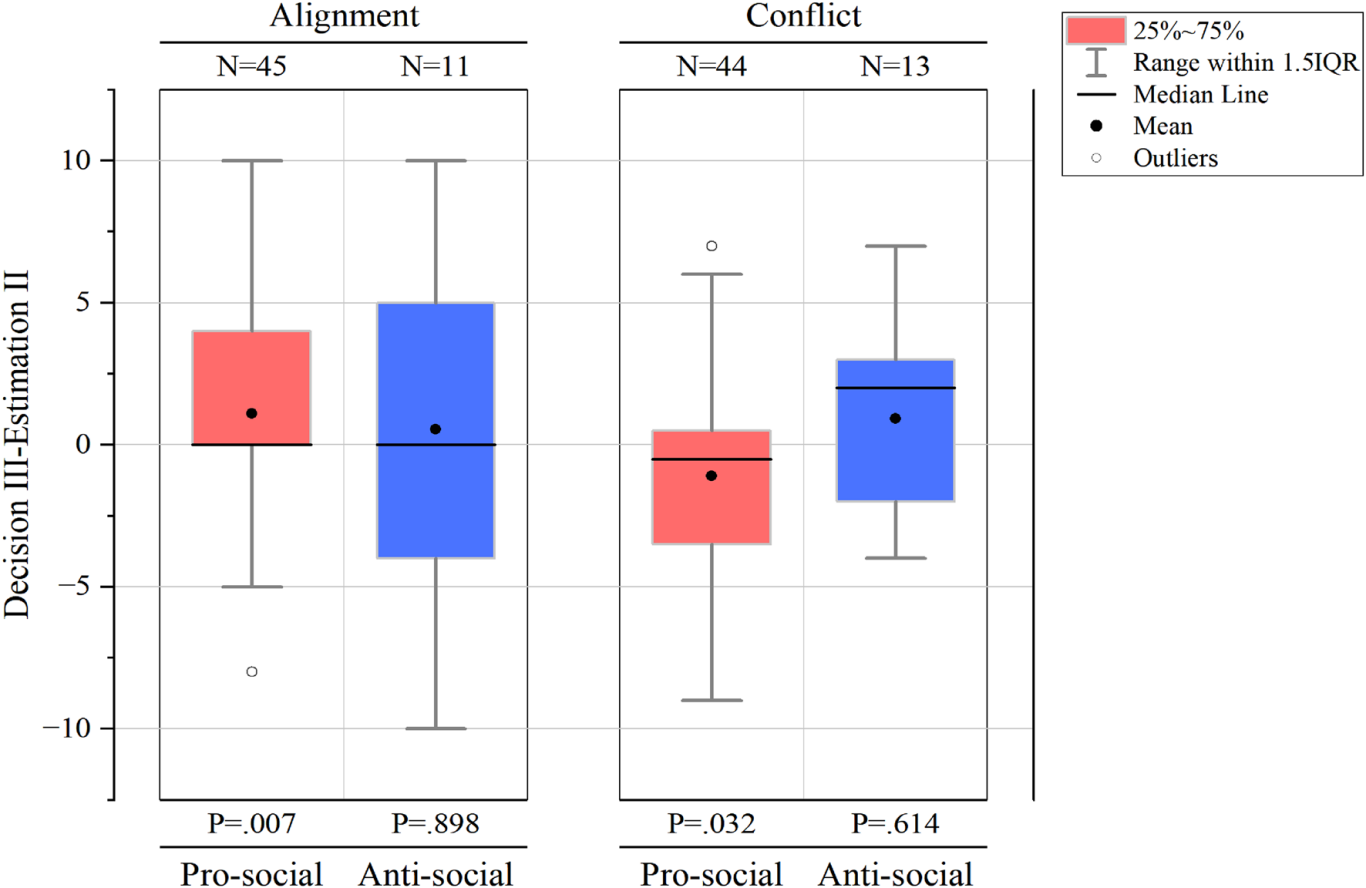
Comparison of Decision III and Estimation II. The left panel shows the differences between Decision III and Estimation II (Decision III–Estimation II) under alignment framework. When facing pro-social behaviors, Decision IIIs significantly exceeded Estimation IIs by 1.11 on average (Mann–Whitney $\hbox {U}=730.0$, $p=.007$); when facing anti-social behaviors, Decision IIIs exceeded Estimation IIs by 0.54 (Mann–Whitney $\hbox {U}=58.5$, $p=.898$). The right panel demonstrates the differences under conflict framework. When facing pro-social behaviors, Decision IIIs were significantly smaller than Estimation IIs by 1.09 on average (Mann–Whitney $\hbox {U}=716.5$, $p=.032$); when facing anti-social behaviors, Decision IIIs exceeded Estimation IIs by 0.93 (Mann–Whitney $\hbox {U}=74.0$, $p=.614$). The solid black dots in the boxes represent means, the black lines represent medians, the white dots outside the boxes represent outliers, and the error bars represent range within 1.5IQR.

Figure 3 presents the differences between Decision III and Estimation II. When Player I was pro-social and the payoffs of Player I and III were aligned, Decision III and Estimation II differed significantly with Decision III exceeding Estimation II by 1.11 on average, indicating that Player III’s willingness to reward exceeded social anticipation as they could benefit from reward under the alignment framework, providing evidence for self-serving reward.

We also found significant difference when Player I was pro-social and Player I and III’s interests were in conflict. However, results suggested that Decision III was smaller than Estimation II by 1.09 on average, indicating that Player III’s rewarding level exceeded social anticipation, that Player III sacrificed individual interest to reward Player I’s good deed. Such altruistic third-party reward was beyond our expectation. The results revealed that third-party were more generous and altruistic than we believed, and were willing to incur personal costs to reward altruistic first-party.

When Player I acted anti-socially, no significant difference between Decision III and Estimation II under either alignment or conflict framework was observed. But according to the mean values, when Player I and III were under alignment framework, Decision III exceeded Estimation II by 0.54, indicating that third-party’s punishment level fell short of social anticipation as punishment diminish their own payoff. Under the conflict framework, Decision III exceeded Estimation II by 0.93, revealing that Player III punished even harsher than society anticipated as severe punishment brought about additional profit. These results provided evidence for third-party’s self-serving punishment, and third-party’s tendency towards self-serving punishment was more obvious when their interests conflicted with the wrong-doer.

We have found solid evidence for third-party’s self-serving reward and punishment. Results revealed that even though third-party generally tended to reward altruism and punish selfishness, sometimes they intentionally avoided the opportunity to do so, or deviated from the reward or punishment level deemed “legitimate” by social anticipation for fear of sacrificing personal gain. When interests aligned, they were generous rewarders and reluctant punishers, and when interests conflict, they were severe punishers. However, we also found that third-party could be more generous and altruistic than we originally believed. Their willingness to reward good deeds at their own expense exceeded social anticipation, which provided evidence for third-party’s altruistic reward.

#### *Result 2*

The experimental results are partially consistent with Hypothesis 2. There is a significant difference between third-party decisions and social anticipation when pro-social behaviors are observed: under the alignment framework, Y-values decided by third-party are significantly higher than social anticipation, revealing third-party’s self-serving reward; however, under the conflict framework, Y-values decided by third-party are significantly lower than social anticipation, revealing altruistic reward. When anti-social behaviors are observed, no significant difference between third-party decisions and social anticipation is found, but the mean Y-values indicate that third-party’s punishment level falls short of social anticipation when interests align and exceeds social anticipation when interests conflict. Third-party’s punitive decisions deviate from social anticipation for the sake of more personal gain, providing evidence for self-serving punishment.

### The amplification effect

We predict in Hypothesis 3 that a small difference in decisions by Player I in stage 1 may result in a large difference in payoffs in stage 2, depending on whether Player III’s payoff is aligned or in conflict with Player I’s payoff. To verify this hypothesis, we performed a one-way ANOVA on $\hbox {P}_I$ in stage 2. We categorized $\hbox {P}_I$ into four groups: (1) in stage 1, Player I was pro-social and in stage 2, Player III and Player I were under alignment framework; (2) Player I was anti-social, and Player III and Player I were under alignment framework; (3) Player I was pro-social, and Player III and Player I were under conflict framework; (4) Player I was anti-social, and Player III and Player I were under conflict framework. The results revealed that there was a statistically significant difference in $\hbox {P}_I$ between the four groups ($\hbox {F}(3, 109)=23.103$, $p<.001$, one-way ANOVA test).

According to post hoc comparisons using the LSD test, when Player III and I were under alignment framework in stage 2, $\hbox {P}_I$ was significantly higher when Player I acted pro-socially than anti-socially($p=.060$, 95%$\hbox {C.I.}= [-.21,10.58]$). When Player III and I were under conflict framework in stage 2, $\hbox {P}_I$ was also significantly higher when Player I acted pro-socially than anti-socially($p<.01$, 95%$\hbox {C.I.}= [5.65,15.77]$). Interestingly, comparing group (1) with group (4), we noticed that the difference between the mean values of $\hbox {P}_I$ amounted to 20 ($p<.01$, 95%$\hbox {C.I.}= [14.80, 24.90]$), providing solid evidence that even though first-party’s decisions exhibited only a small difference, due to alignment or conflict of interests, third-party amplified the implication of this small-stake decision to justify reward and punishment, in this process seeking more personal gain and leading to a huge difference in $\hbox {P}_I$ in stage 2.

These results are consistent with our prediction. We can draw the following conclusion:

#### *Result 3*

The experimental results concur with Hypothesis 3. Third-party amplify the implication of first-party’s small-stake decisions due to alignment or conflict of interests, which results in a significant difference in first-party’s payoffs. Under both alignment and conflict frameworks, the payoffs of pro-social first-party are significantly higher than that of anti-social first-party. Noticeably, when interests align, the payoffs of pro-social first party exceed that of anti-social first party when interests conflict by 20. This significant difference amounts to two-thirds of the highest possible payoff, providing evidence for the amplification effect.

#### Comparison of Y-values

**Figure 4:**
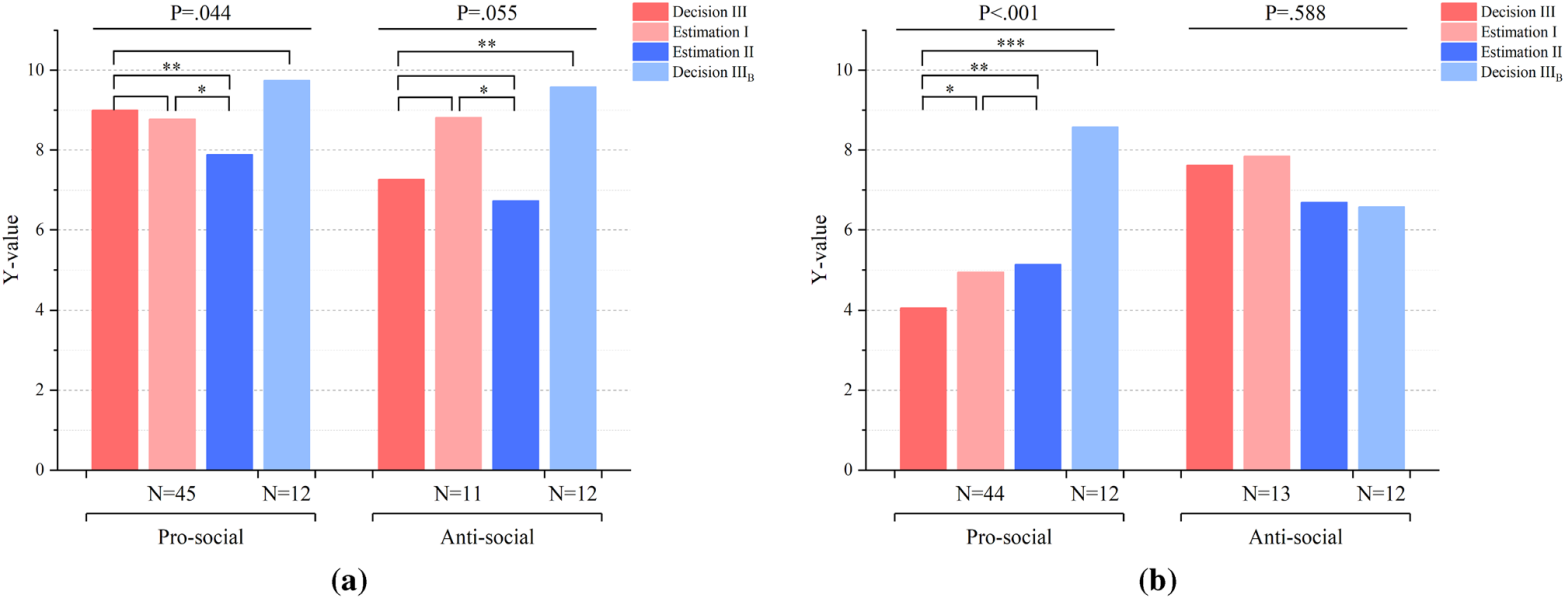
Comparison of four Y-values: Decision III, Estimation I, Estimation II and Decision III$_B$. In (**a**), the interests of Player I and III were aligned. When facing pro-social behaviors, the four Y-values were significantly different at the .05 level ($\hbox {F}(3,143)=2.775$, $p=.044$, one-way ANOVA test). Post hoc comparisons using Fisher’s LSD indicated that Decision IIIs were significantly larger than Estimation IIs ($p=.028$, 95%$\hbox {C.I.}= [.1227, 2.0995]$), and Estimation Is were significantly higher than Estimation IIs ($p=.078$, 95%$\hbox {C.I.}= [-.0995, 1.8773]$). When facing anti-social behaviors, the four Y-values were significantly different at the .1 level ($\hbox {F}(3,41)=2.756$, $p=.055$, one-way ANOVA test), with Estimation Is significantly higher than Estimation IIs ($p=.077$, 95%$\hbox {C.I.}= [-.2343, 4.4161]$) and Decision IIIs significantly lower than Decision III$_B$s ($p=.047$, 95%$\hbox {C.I.}= [-4.5868, -.0344]$). In (**b**), the interests of Player I and III were in conflict. When facing pro-social behaviors, the four Y-values were significantly different at the .01 level ($\hbox {F}(3,140)=9.888$, $p<.001$, one-way ANOVA test), with Decision IIIs significantly lower than Estimation Is ($p=.098$, 95%$\hbox {C.I.}= [-1.9896,.1714]$), Estimation IIs ($p=.048$, 95%$\hbox {C.I.}= [-2.1714, -.0104]$), and Decision III$_B$s ($p<.001$, 95%$\hbox {C.I.}= [-6.1883, -2.8874]$); when facing anti-social behaviors, no significant difference was found ($\hbox {F}(3,47)=.649$, $p=.588$, one-way ANOVA test).

We also validated our previous prediction for the four Y-values: Decision III$_B$, Decision III, Estimation I, Estimation II. Below Fig. 4 shows the mean of each Y-value and the result of one-way ANOVA and Fisher’s LSD test in each scenario. We sorted the four Y-values in Table 3 and compared them with our previous prediction in Table 2.

**Table 3:** Comparison of Y-values.

STAGE 2: Framework	STAGE 1: Behavior	Decision III & Estimation I & Estimation II	Decision III & Decision III$_B$
Alignment $\hbox {P}_{III}=20+\hbox {Y}$; $\hbox {P}_I=3\hbox {Y}$	Pro-social	Decision III $>$ Estimation I > Estimation II	Decision III$_B>$ Decision III
	Anti-social	Estimation I > Decision III > Estimation II	Decision III$_B>$ Decision III
Conflict $\hbox {P}_{III}=20+\hbox {Y}$; $\hbox {P}_I=30-3\hbox {Y}$	Pro-social	Estimation II > Estimation I > Decision III	Decision III$_B>$ Decision III
	Anti-social	Estimation I > Decision III > Estimation II	Decision III > Decision III$_B$

Comparison of Decision III, Estimation I, Estimation II and Decision III$_B$ in four different scenarios: interests of Player I and III were aligned, and Player I was either pro-social or anti-social; and interests of Player I and III were in conflict, and Player I was either pro-social or anti-social.

When Player III and I were under alignment framework, and Player I acted pro-socially, as we had expected, all four mean values were close to 10, with Estimation II slightly smaller and Decision III$_B$ slightly larger than the other two. When Player I acted anti-socially, the result was exactly what we had expected, that Decision III’s punishment level was lesser than social anticipation, revealing third-party’s self-serving punishment; Y-value estimated by Player I was even larger than Decision III, and Decision III$_B$ was larger than Decision III.

When Player III and I were under conflict framework, and Player I’s pro-social behavior was observed, surprisingly, Decision III was much lower than we had anticipated, demonstrating altruistic reward. When Player I’s anti-social behavior was observed, the result was generally in line with our expectation except for Estimation I, which presented the largest Y-value, indicating that Player I expected a harsh punishment. This shows that when first-party commit selfish behaviors, they know they are acting against social norms and are fully aware of the consequence.

## Discussion

We provide evidence that the decisions of third-party to reward or punish are often self-serving. While third-party are seemingly willing to incur a cost to reward altruism and punish selfishness, in secret, they are reluctant to do so as their personal interests are harmed in this process. Without revealing their true self-serving belief, they deviate from social anticipation for “legitimate” third-party responses. Specifically, when they can gain profit out of third-party decisions, third-party’s reward and punishment level exceed social anticipation of a “legitimate” third-party response to pro-social or anti-social behaviors; however, when their interests diminish with increasing intensity of reward and punishment, third-party tend to shirk the responsibility to reward and punish, or their reward and punishment level falls short of public’s anticipation of a “legitimate” third-party response. While^23^ also reveals third-party’s reluctance to punish, it fails to unravel the intrinsic motivations behind it. Jordan et al.^22^ also probe into third-party’s unwillingness to punish, but from the scope of the perception of behaviors, that punishment is perceived as a weaker signal for cooperation and trustworthiness. Our study explains this reluctance of third-party from the perspective of self-serving belief and enhances the understanding of third-party behaviors.

Our work also highlights the need to better understand the deeper motives of third-party behaviors. From the findings of^17–23,33,36,37^, we can categorize the incentives that influence third-party decisions into intrinsic and extrinsic motives: intrinsic motives include third-party’s perception of social norms such as altruism, their perception of actions such as reward and punishment, and also their desire to be valued positively; extrinsic motives include payoff scheme and decision transparency. Our study enriches existing literature on incentives of third-party behaviors by centering on another intrinsic motive, which is third-party’s self-serving belief.

This study also brings up the issues of the interest relationship between third-party and first-party, and how this interest relationship influences third-party behaviors. This is realized through two different payoff schemes: alignment and conflict. Under the alignment framework, the payoffs of third-party and first-party are positively correlated whereas under the conflict framework, their interests are in conflict, one gains at the cost of the other. Our design of the two payoff frameworks combines and further develops the payoff schemes of “profit-seeking” versus “costly”, and “profitable” versus “non-profitable” used in^23,39^. By adopting the alignment and conflict frameworks, our study provides important insights into how third-party’s willingness to reward and punish differ depending on their interest relationship with the first-party.

More broadly, our findings emphasize the significance of comprehending the underlying motives behind various third-party behaviors to gain a more precise understanding of their potential impact on society. While the willingness to incur costs to reward norm adherence and punish norm violation appears to be a prevalent phenomenon in society, our study indicates that third-party are secretly self-serving. Their motives underlying such apparent preferences and the joint influence of different motives are extremely subtle and complex. Therefore, investigating how and to which extent third-party’s self-serving belief impacts behaviors can shed light on the explanation of real-life sanctioning outcomes in complex scenarios.

## Methods

### Subject and method

All the experimental sessions were conducted at the Behavioral and Experimental Social Science Laboratory (BESSL) at Beijing Foreign Studies University (BFSU) in September and October 2022. The Ethical Review Committee of the Behavioral and Experimental Social Science Laboratory at Beijing Foreign Studies University approved the study (Reference No. 2022L009). The computerized experiment was designed using the software program Z-tree^42^.

The study was carried out in accordance with the relevant guidelines and regulations of Beijing Foreign Studies University for studies with human participants. A total of 483 subjects were recruited via a web-based recruitment system that draws on a large pool of potential student subjects in BFSU. The number of subjects who participated in each treatment is listed below in Table 4. Each of the 483 subjects who participated took part in one treatment only.

**Table 4:** Subject.

Treatment	OBS
T1: A–A	87
T2: A–C	84
T3: C–A	81
T4: C–C	87
T5: B–A–A	36
T6: B–A–C	36
T7: B–C–A	36
T8: B–C–C	36
Total	483

Among all the subjects, a total of 83% of the subjects were female, and the median age was 20. 64% of the subjects were economic majors, and others majored in information management, languages, education, international relations and journalism. For social income, 54% had a weekly income between RMB 300 to RMB 700, 25% had a weekly income lower than RMB 300 and 21% had a weekly income over RMB 700.

All subjects gave their informed consent before participation. After that, subjects received written instructions that were subsequently read aloud to promote understanding. Then, subjects completed the experimental procedure following instructions shown stage-by-stage on computer screens.  Screenshots of the experimental stages are provided in the Supplementary File. Subjects earned tokens, convertible into RMB at the end of the experiment($1\,\hbox {token}=1\,\hbox {RMB}$) in amounts determined by the outcome of the experiment. The eight treatments were carried out serially, and each treatment included three rounds. The computer randomly selected one round, the payoff of which was output as the final payoff. Subjects gained RMB 27.6 on average over the 15-min experiment, plus a show-up fee of RMB 10.

To analyze the results, we applied the Mann-Whitney U test, ANOVA and LSD. The Mann-Whitney U test, also known as the Wilcoxon Rank Sum Test, is a non-parametric statistical test used to compare two samples or groups. It assesses whether two samples are likely to derive from the same population, and essentially asks whether these two populations have the same shape with regard to their data. In this study, we employed the Mann-Whitney U test to test: (1) the difference between third-party’s decisions when the first-party they face is either pro-social or anti-social; (2) the difference between third-party’s decisions in main treatment groups and in benchmark groups; (3) the difference between third-party’s decisions and society’s anticipation of reward and punishment that should be exerted on first-party. The results of the Mann Whitney U test showed accordingly: (1) third-party’s decisions differed significantly in the face of either pro-social or anti-social first-party; (2) third-party’s decisions differed significantly in response to X-values made by first-party or randomly assigned by computers, demonstrating third-party reward and punishment; (3) Third-party’s decisions significantly deviated from social anticipation, revealing both self-serving reward and altruistic reward. We also used one-way ANOVA, which is a statistical tool analyzing the differences among means of different groups. We probed into the differences between payoffs of Player Is in different scenarios, and we applied Fisher’s LSD to create confidence intervals for all pairwise differences, providing solid evidence for the amplification effect. We also used one-way ANOVA and LSD to test the difference between Decision III, Estimation I, Estimation II and Decision III$_B$.

### Instructions

#### General instructions

Thank you for participating in this experiment! We will pay a participation fee of 10 RMB for you. Please read the following instructions carefully, and if you have any questions, feel free to ask us. Please do not communicate with others during the experiment. Thank you for your cooperation.

Matching rules: Before the experiment begins, you will be randomly assigned an ID number (Player I, Player II, or Player III) to represent your Player role. You will randomly form a group of three people with other 2 participants with different roles to complete this experimental task.

Payoff rules: The payoff you receive will be converted from your tokens earned during the experiment. Your payoff is converted following the rule: $1~\hbox {token} = 1~\hbox {RMB}$.

This experimental task consists of several stages. In order to ensure the smooth running of the experimental task, the rules of each stage will be informed in turn.

#### STAGE 1 Instructions

In this stage, only Player I will make a decision that will determine the payoffs of Player I and fellow Player II in stage 1. Player I needs to decide on the value of X (0 or 1). Player II can only accept, not reject.

Stage 1 payoff rules:

- $$\hbox {P}_I= 2 + \hbox {X}$$
- $\hbox {P}_{II} = 3\hbox {X}$ (under the Alignment framework; under the Conflict framework, $\hbox {P}_{II} = 3 - 3\hbox {X}$)

#### STAGE 2 Instructions

In this stage, the group composition of Player I, Player II and Player III remains the same as in stage 1. We will inform Player III of the decision made by fellow Player I in stage 1.

In this stage, only Player III will make a decision that will determine the payoffs of Player III and fellow Player I in stage 2. Player III needs to decide on the value of Y (an integer between 0 and 10). Player I can only accept, not reject.

Stage 2 payoff rules:

- $$\hbox {P}_{III} = 20 + Y$$
- $\hbox {P}_I= 3Y$ (under the Alignment framework; under the Conflict framework, $\hbox {P}_I= 30 - 3\hbox {Y}$)

#### STAGE 3 Instructions

In this stage, the group composition of Player I, Player II, and Player III remains the same as in stage 1. Player I and Player II need to make the following estimations.

Player I: We will not inform Player I of the decisions made by Player III in stage 2. At this point Player I needs to estimate the Y-value of Player III’s decision in stage 2. The closer the estimated Y-value of Player I is to the true value, the more tokens will be rewarded. The specific bonus rule is as follows:

Assuming that Player I’s estimation of Y-value is G and Player III’s actual decision of Y-value is T, Player I receives a $\hbox {bonus} = 10*[1-((T-G)/10)^2]$ (Hint: Player I receives the highest bonus when Player I’s estimation G equals Player III’s actual decision T).

Player II: We will not inform Player II of the decisions made by Player I and Player III in stage 1 and 2 respectively. At this point, Player II needs to make two estimations as follows:

First, Player II needs to estimate the decision made by Player I in stage 1. If the estimation is accurate, Player II will be rewarded with 5 tokens. If the estimation is incorrect, Player II will receive 0 token.

Second, Player II needs to estimate when the X-value of Player I’s decision in stage 1 is 0 or 1 respectively, the Y-value of Player III’s decision in stage 2. The closer the estimated Y-value of Player II is to the true value, the more tokens will be rewarded. The specific bonus rule is as follows:

Assuming that Player II’s estimation of Y-value is G and Player III’s actual decision of Y-value is T, Player II receives a $\hbox {bonus} = 10*[1-((T-G)/10)^ 2]$ (Hint: Player II receives the highest bonus when Player II’s estimation G equals Player III’s actual decision T).

Stage 3 payoff rules:

- $\hbox {P}_I = \hbox {bonus}$ for estimation of Player III’s decision
- $\hbox {P}_{II} = \hbox {bonus}$ for estimation of Player I’s Decision + bonus for estimation of Player III’s Decision

The total payoff of this experiment task is:

- Total $\hbox {P}_I = \hbox {Stage 1 P}_I + \hbox {Stage 2 P}_I + \hbox {Stage 3}$ bonus for estimation of Player III’s decision
- Total $\hbox {P}_{II} = \hbox {Stage 1 P}_{II} + \hbox {Stage 3}$ bonus for estimation of Player I’s Decision + Stage 3 bonus for estimation of Player III’s Decision
- Total $$\hbox {P}_{III} = \hbox {Stage 2 P}_{III}$$

#### STAGE 1 Instructions-benchmark groups

In this stage, the computer will generate a random X-value (0 or 1) which will determine the payoffs of Player I and fellow Player II in stage 1.

Stage 1 payoff rules:

- $$\hbox {P}_I= 2 + \hbox {X}$$
- $\hbox {P}_{II} = 3\hbox {X}$ (under the Alignment framework; under the Conflict framework, $\hbox {P}_{II} = 3 - 3\hbox {X}$)

#### STAGE 2 Instructions-benchmark groups

In this stage, the group composition of Player I, Player II and Player III remains the same as in stage 1. We will inform Player III of the random X-value (0 or 1) generated by the computer and the payoffs of Player I and Player II in stage 1.)

In this stage, only Player III will make a decision that will determine the payoffs of Player III and fellow Player I in stage 2. Player III needs to decide on the value of Y (an integer between 0 and 10). Player I can only accept, not reject.

Stage 2 payoff rules:

- $$\hbox {P}_{III} = 20 + \hbox {Y}$$
- $\hbox {P}_I= 3\hbox {Y}$ (under the Alignment framework; under the Conflict framework, $\hbox {P}_I= 30 - 3\hbox {Y}$)

#### STAGE 3 Instructions-benchmark groups

In this stage, the group composition of Player I, Player II, and Player III remains the same as in stage 1. Player I and Player II need to make the following estimations.

Player I: We will not inform Player I of the decisions made by Player III in stage 2. At this point Player I needs to estimate the Y-value of Player III’s decision in stage 2. The closer the estimated Y-value of Player I is to the true value, the more tokens will be rewarded. The specific bonus rule is as follows:

Assuming that Player I’s estimation of Y-value is G and Player III’s actual decision of Y-value is T, Player I receives a $\hbox {bonus} = 10*[1-((T-G)/10)^2]$ (Hint: Player I receives the highest bonus when Player I’s estimation G equals Player III’s actual decision T).

Player II: We will not inform Player II of the random X-value (0 or 1) generated by the computer and Player III’s decision in stage 1 and 2 respectively. At this point, Player II needs to make two estimations as follows:

First, Player II needs to estimate the random X-value (0 or 1) generated by the computer in stage 1. If the estimation is accurate, Player II will be rewarded with 5 tokens. If the estimation is incorrect, Player II will receive 0 token.

Second, Player II needs to estimate when the random X-value (0 or 1) generated by the computer in stage 1 is 0 or 1 respectively, the Y-value of Player III’s decision in stage 2. The closer the estimated Y-value of Player II is to the true value, the more tokens will be rewarded. The specific bonus rule is as follows:

Assuming that Player II’s estimation of Y-value is G and Player III’s actual decision of Y-value is T, Player II receives a $\hbox {bonus} = 10*[1-((T-G)/10)^2]$ (Hint: Player II receives the highest bonus when Player II’s estimation G equals Player III’s actual decision T).

Stage 3 payoff rules:

- $\hbox {P}_I = \hbox {bonus}$ for estimation of Player III’s decision
- $\hbox {P}_{II} = \hbox {bonus}$ for estimation of random X-value + bonus for estimation of Player III’s Decision

The total payoff of this experiment task is:

- Total $\hbox {P}_I = \hbox {Stage 1 P}_I + \hbox {Stage 2 P}_I + \hbox {Stage 3}$ bonus for estimation of Player III’s decision
- Total $\hbox {P}_{II} = \hbox {Stage 1 P}_{II} + \hbox {Stage 3}$ bonus for estimation of Player I’s Decision + Stage 3 bonus for estimation of Player III’s Decision
- Total $$\hbox {P}_{III} = \hbox {Stage 2 P}_{III}$$

### Additional analysis: the learning effect

#### Distribution of decision III

**Figure 5:**
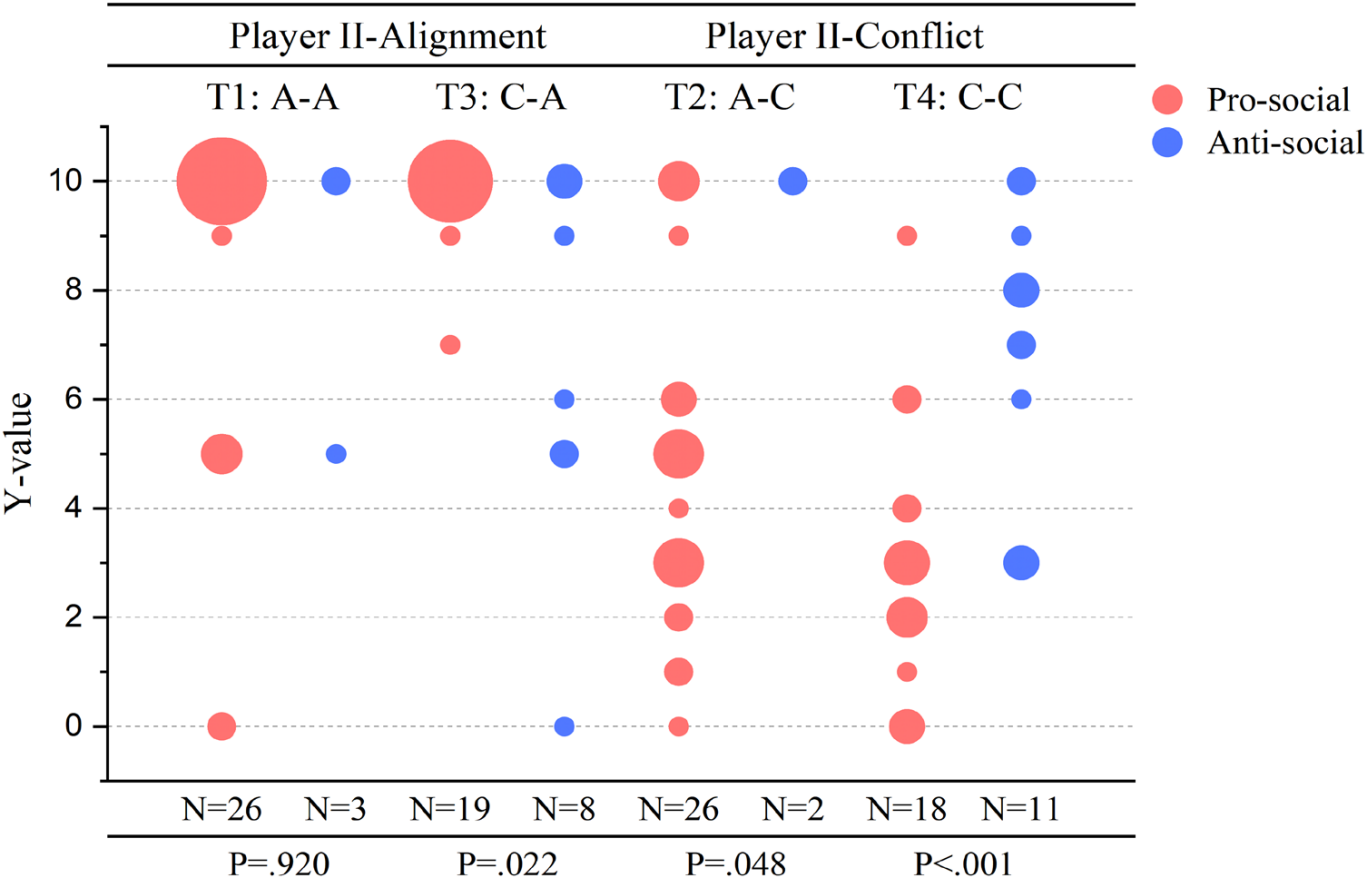
Comparison of third-party’s reward and punishment decisions. Significant differences between third-party’s responses toward pro-social and anti-social behaviors were found in treatment 2, 3, and 4. Each dot represents a Y-value decided by Player III in each treatment, either facing pro-social or anti-social Player I, and the size of the dots corresponds to the occurrence of each Y-value in different scenarios. When facing either pro-social or anti-social Player I, decision IIIs differed significantly in treatment 2 (Mann–Whitney $\hbox {U}=4.0$, $p=.048$), treatment 3 (Mann–Whitney $\hbox {U}=33.0$, $p=.022$), treatment 4 (Mann-Whitney $\hbox {U}=22.5$, $p<.001$). But no significant difference was found in treatment 1(Mann–Whitney $\hbox {U}=37.0$, $p=.920$).

In the section of Third-party Reward and Punishment in Results, we analyzed the distribution of Decision III. While Fig. 2 compares Decision IIIs in the face of pro-social and anti-social Player I, we also took into consideration the payoff framework that Player I was subjected to in stage 1. Below Fig. 5 presents the distribution of Decision III in treatment 1–4. We applied the Mann-Whitney U test to compare Decision IIIs when Player I acted either pro-socially or anti-socially. We can conclude that third-party’s responses toward pro-social and anti-social behaviors differed greatly in treatment 2, 3, and 4, but such distinct difference between reward and punishment could not be found in treatment 1, possibly due to the small sample size of anti-social Player I ($\hbox {N}=3$).

#### The learning effect

**Figure 6:**
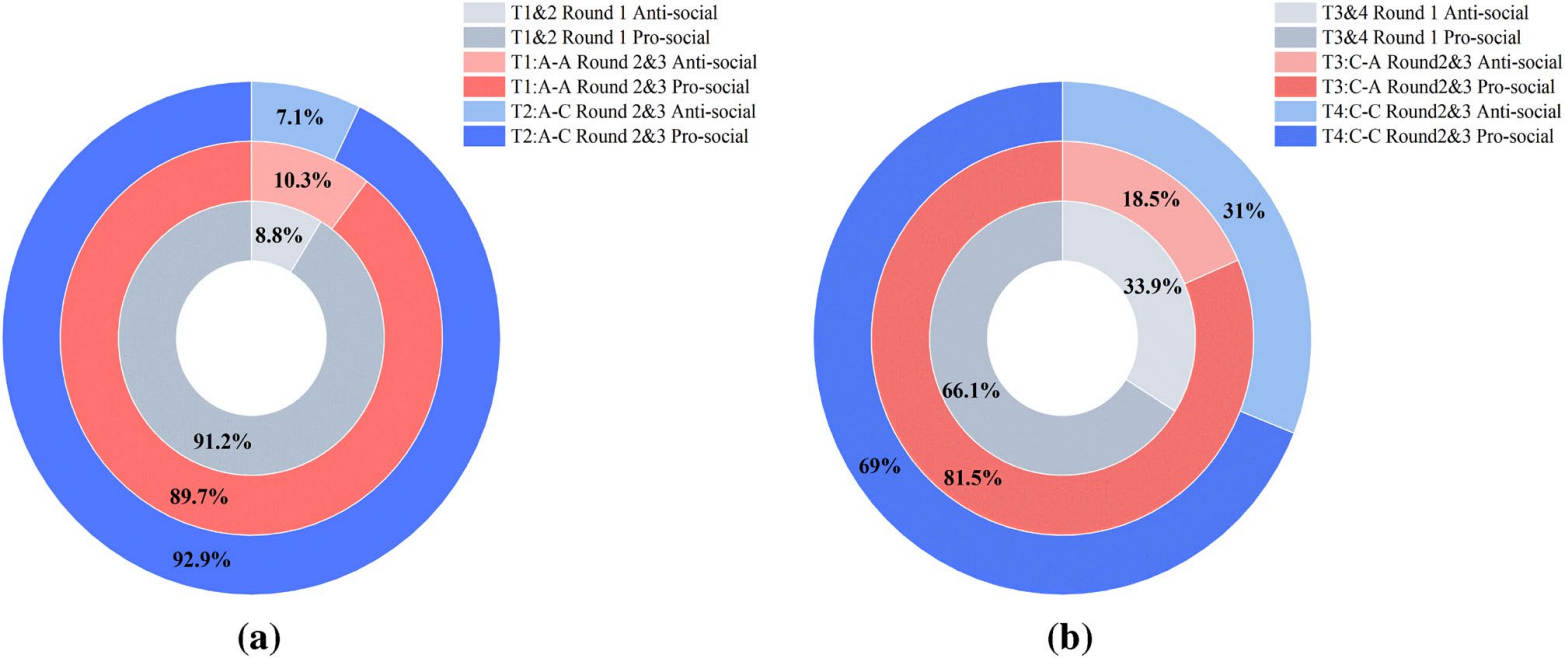
Player Is’ decisions throughout three rounds. (**a**) Distribution of Player I’s decisions when Player I was subject to the alignment framework. The inner circle shows Player Is’ decisions in T1 and T2 in Round 1 ($\hbox {N}=57$). The middle circle shows Player I’s decisions in T1 in Round 2 and 3 ($\hbox {N}=58$). The outer circle shows Player I’s decisions in T2 in Round 2 and 3 ($\hbox {N}=56$). (**b**) Distribution of Player I’s decisions when Player I was subject to the conflict framework. The inner circle shows Player Is’ decisions in T3 and T4 in Round 1 ($\hbox {N}=56$). The middle circle shows Player I’s decisions in T3 in Round 2 and 3 ($\hbox {N}=54$). The outer circle shows Player I’s decisions in T4 in Round 2 and 3 ($\hbox {N}=58$).

We also looked into the experimental results of the second and third round. As subjects got familiar with the experimental content and accumulated experiences, their strategies and decisions differed from that of the first round. Below Fig. 6 demonstrates Player Is’ behaviors.

For Player I’s behaviors, third-party reward and punishment proved to be an efficient way to promote pro-social behaviors. In Fig. 6a, in treatment 1, surprisingly, an increasing portion of Player I chose to act anti-socially, probably because they found out that third-party’s interests aligned with theirs and assumed that severe punishment would not befall them. In treatment 2, the proportion of Player I choosing to act anti-socially diminished, thanks to the sanctions imposed by the third-party. In Fig. 6b, in both treatment 3 and 4, compared with the first round, the occurrence of anti-social behaviors decreased in the second and third round. The influence of third-party was most obvious in treatment 3 where the proportion of selfish Player I diminished by nearly 50%. These findings are consistent with other literature in showing that third-party can sustain conformity with social norms.

As to third-party’s behaviors, they exhibited a stronger tendency for self-serving reward and punishment in the second and third round. After knowing the entire experimental content, they generally decided on higher Y-values. Meanwhile, the discrepancy between Decision III and Estimation II significantly narrowed as Player II gave more and more accurate estimation for third-party responses. We found significant difference between Decision III and Estimation II in the second and third round when stage 2 was under alignment framework and Player I was pro-social(Mann–Whitney $\hbox {U}=3726.5$, $p=.004$), indicating the continuance of self-serving reward in the latter rounds. Though no significant difference suggesting self-serving punishment was observed, we noticed a rise in Y-values when interests conflicted and Player I acted pro-socially, indicating the abatement of third-party’s altruism and the increase of self-serving bias.

### Supplementary Information

Supplementary Information.

## Data Availability

The datasets used and analyzed in this study are available from the corresponding author upon reasonable request.
