# Supplementary material for: Self-serving reward and punishment: evidence from the laboratory
Source: Sci Rep. 2023 Aug 26;13:13997. doi: 10.1038/s41598-023-41256-5 (PMC10460402; doi:10.1038/s41598-023-41256-5)

**Screenshots of experimental set-up**

(Take Treatment 1: A-A as an example)

General Instruction:


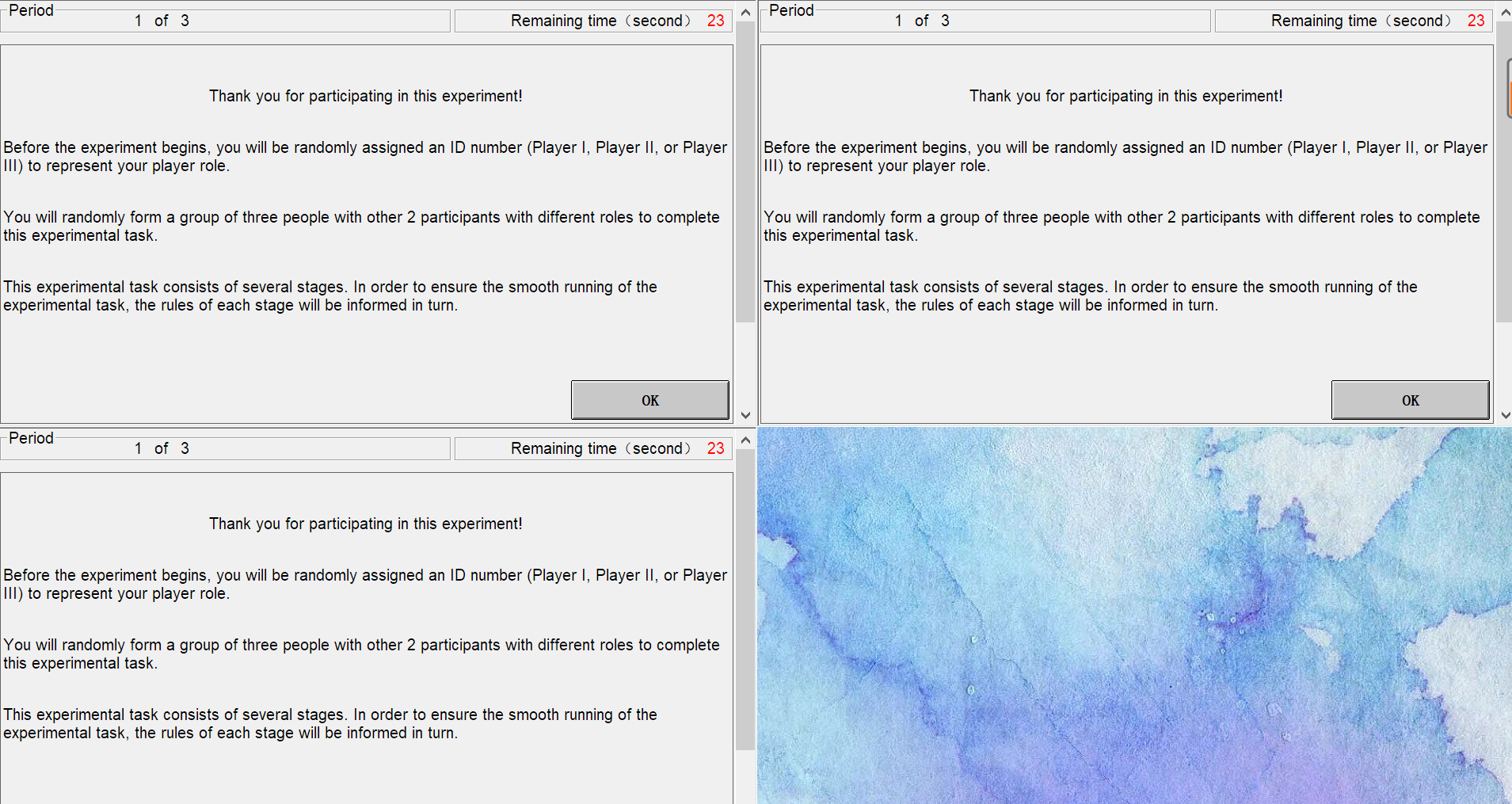


Assign player role of Player I, II or III


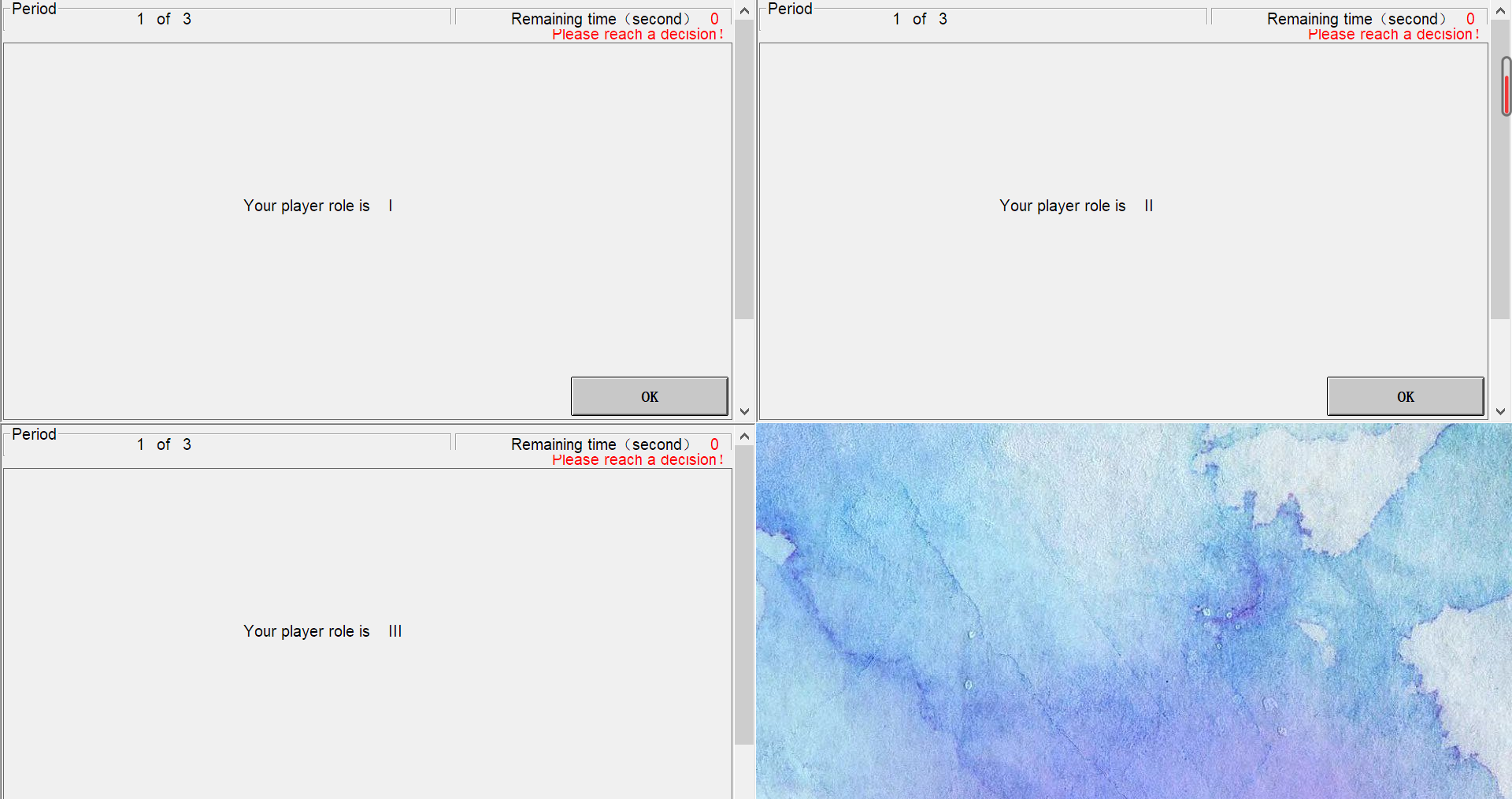


Stage 1:


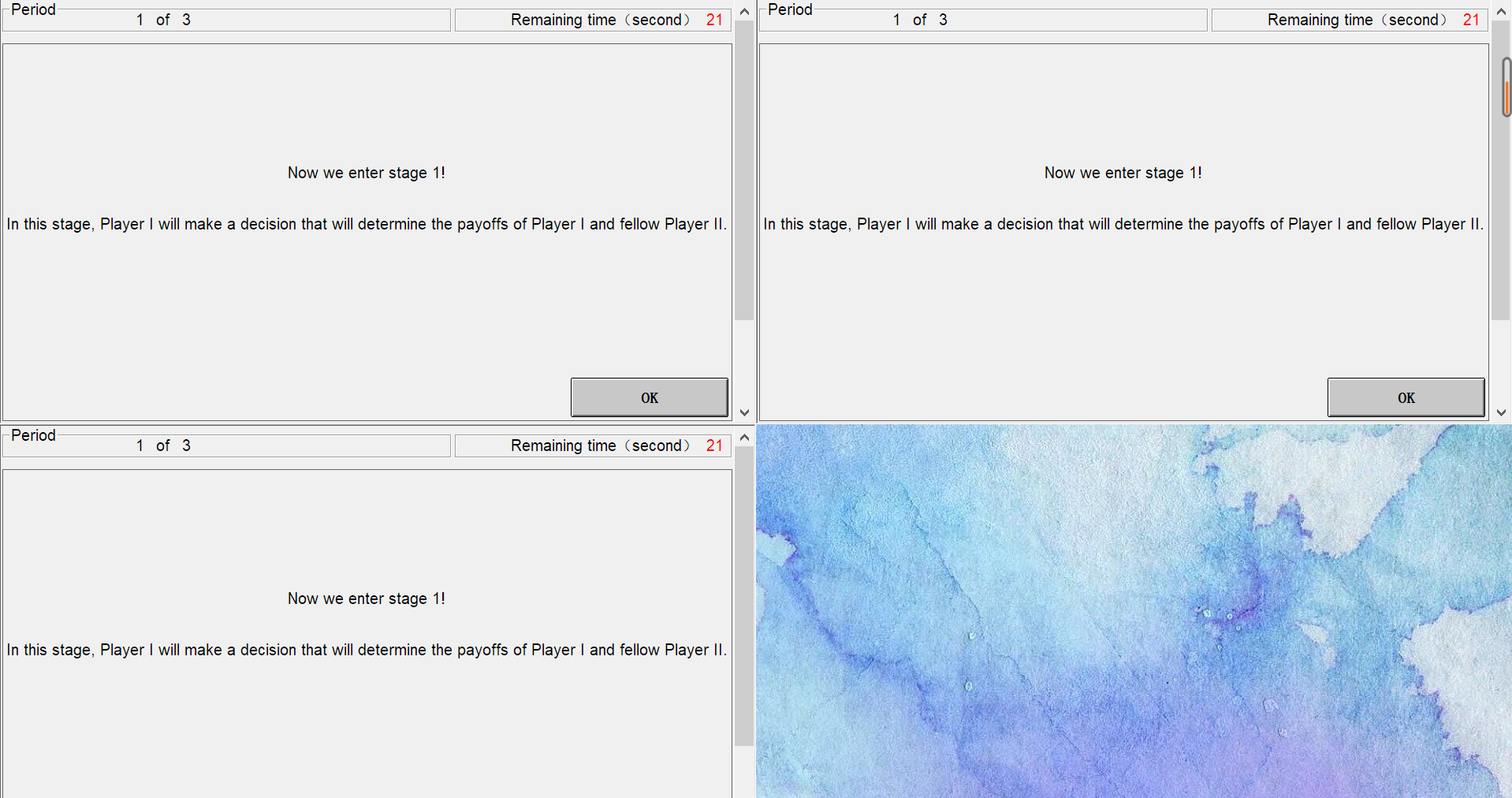

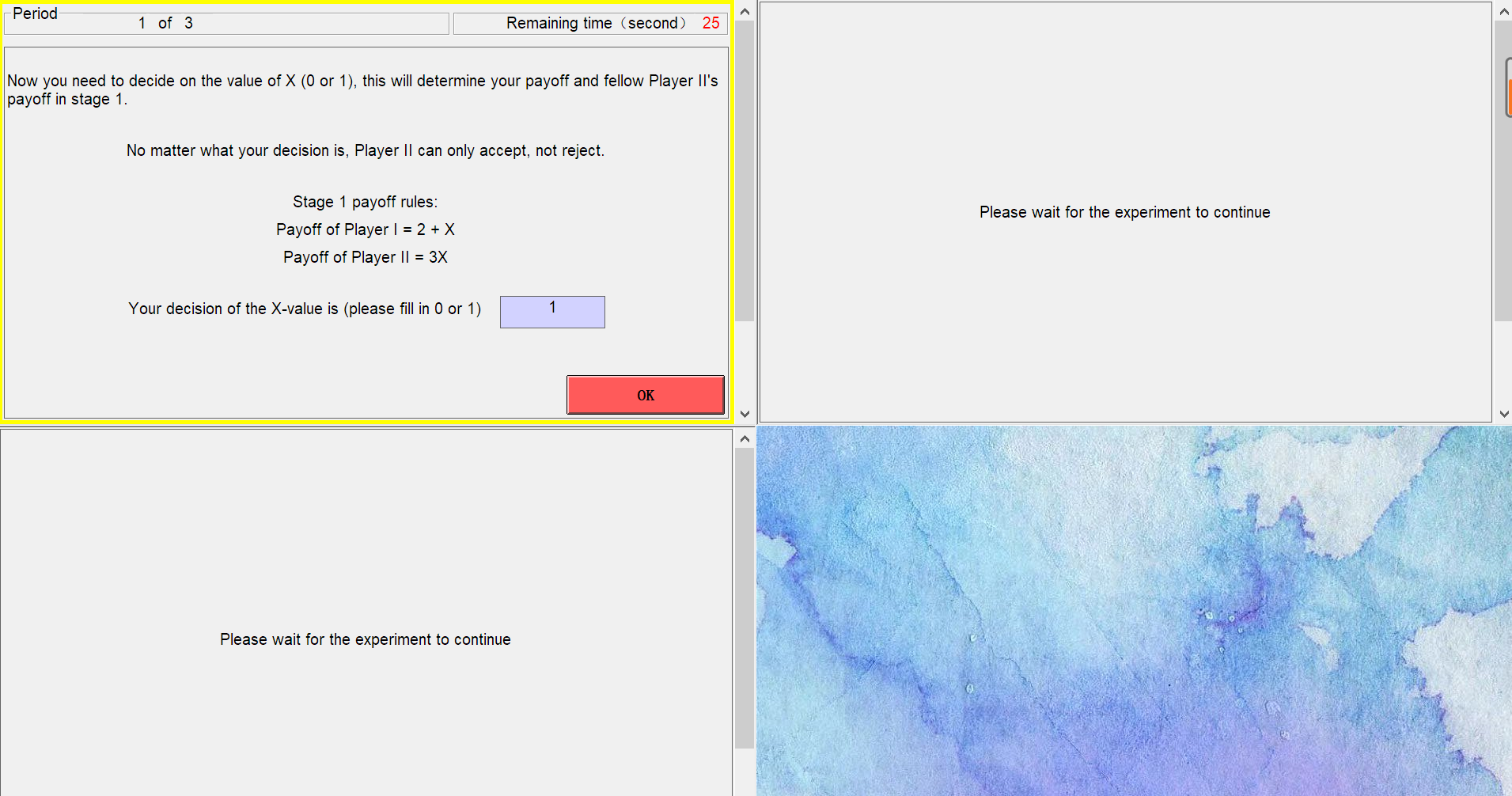

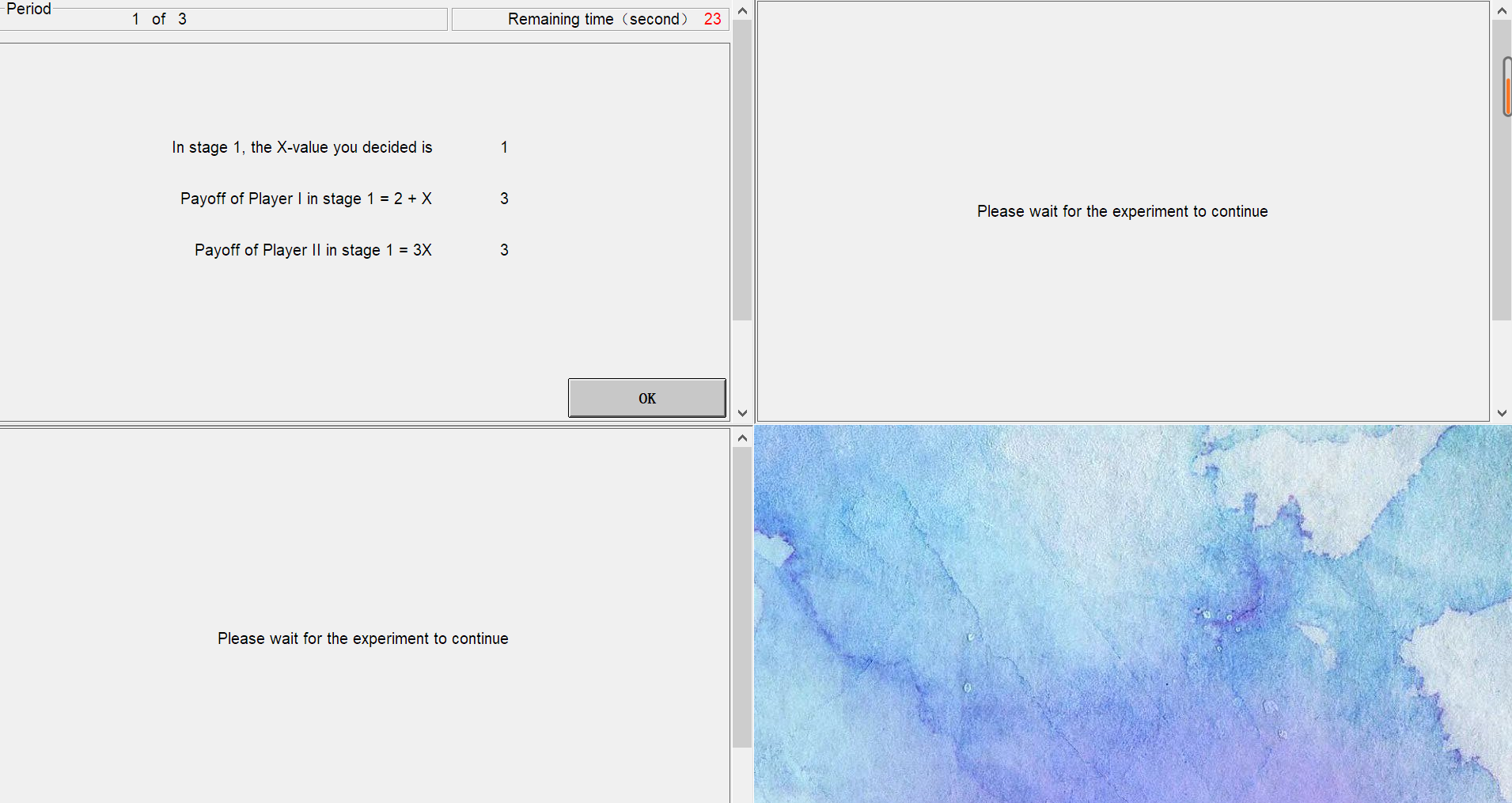


Stage 2:


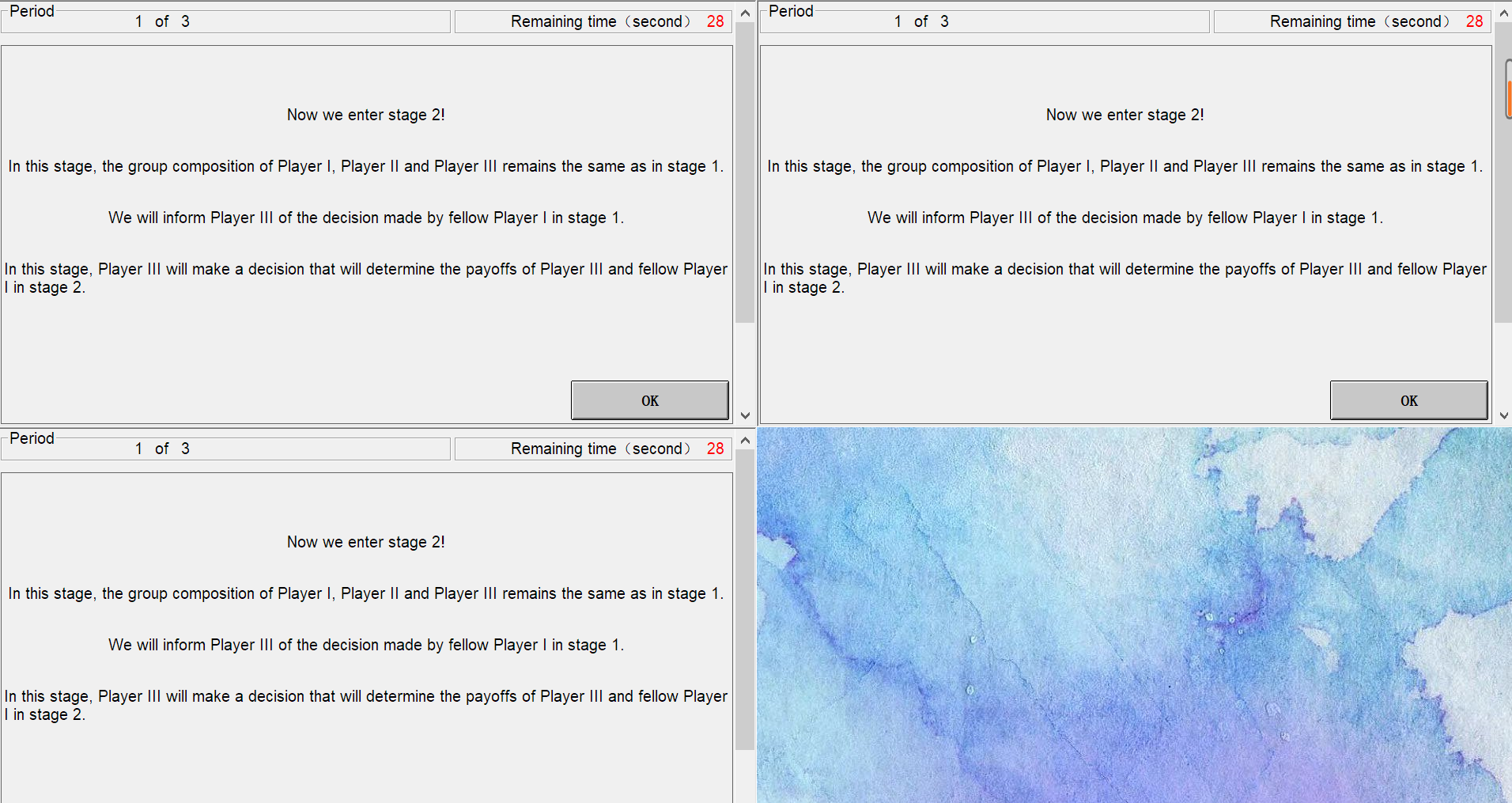

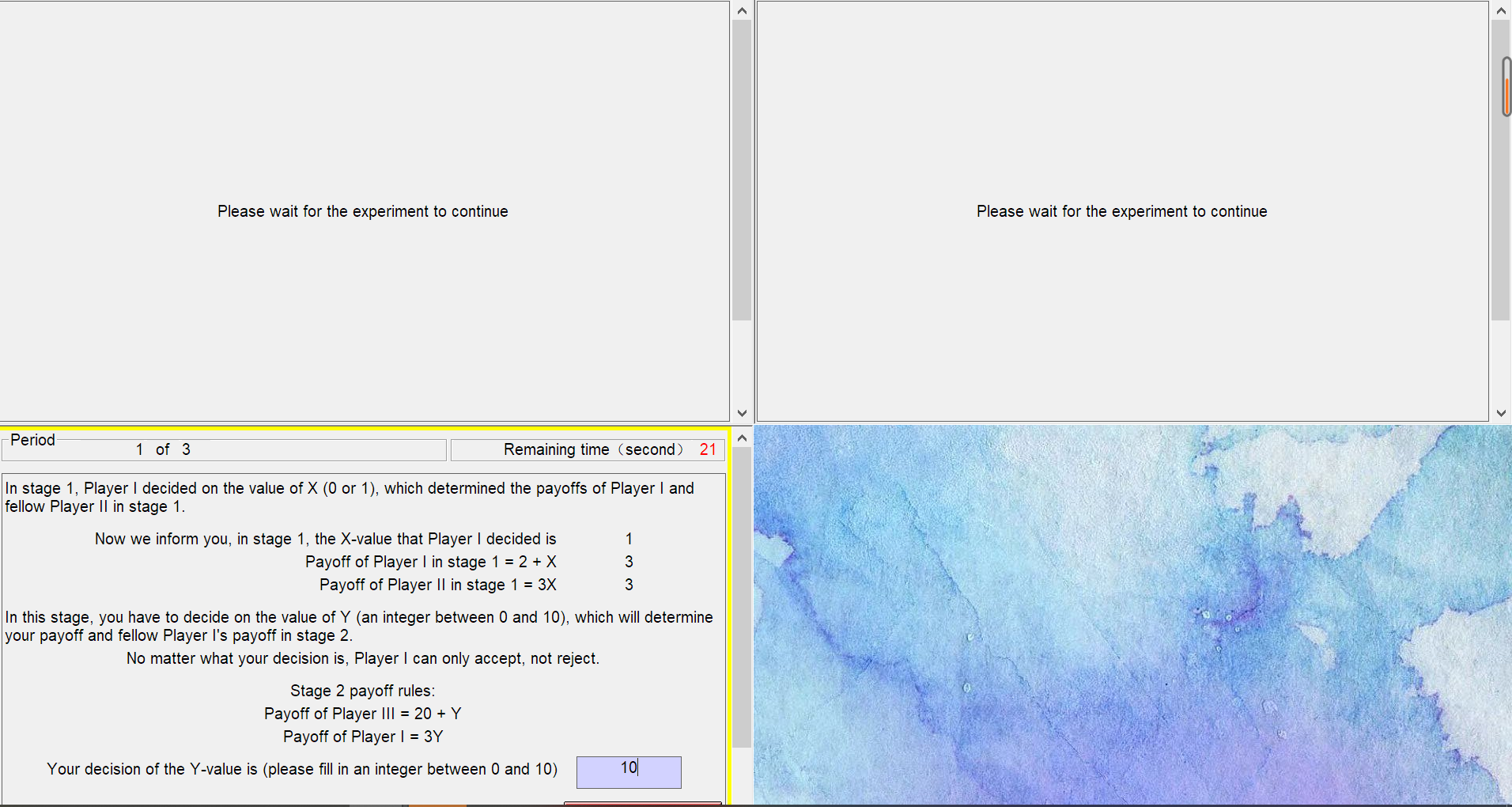

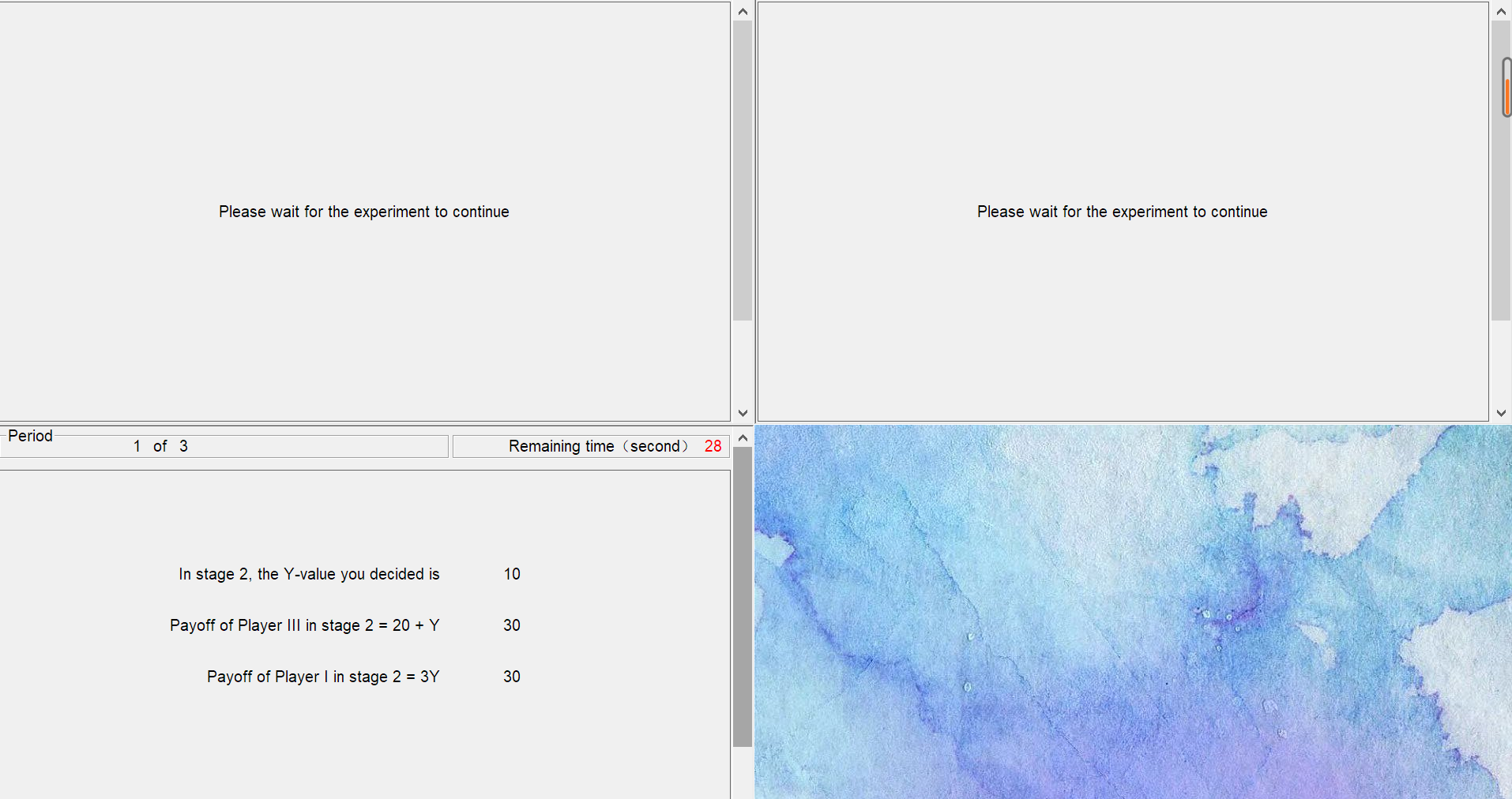


Stage 3 (some interfaces are presented in larger windows for clearer demonstration):


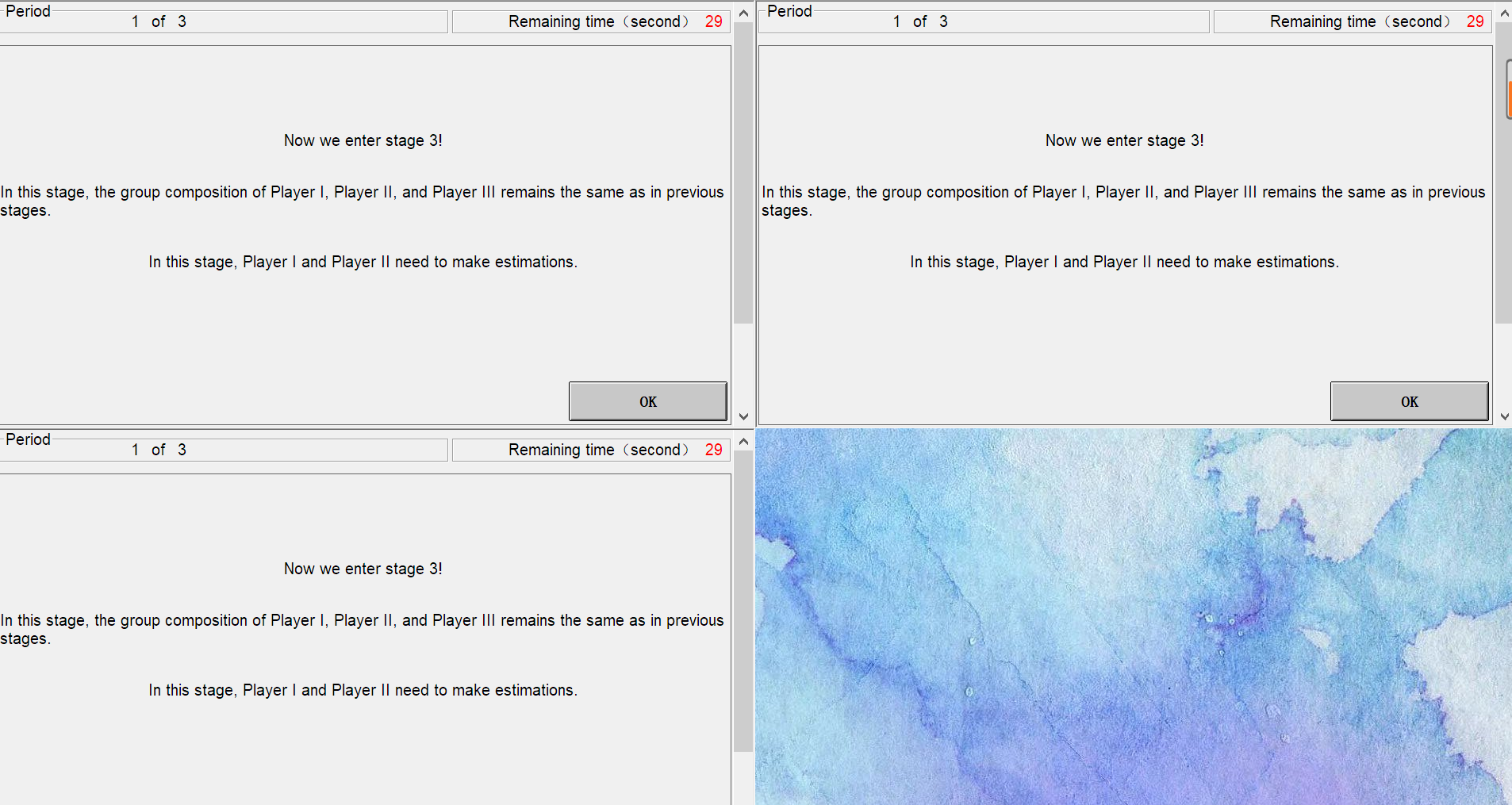

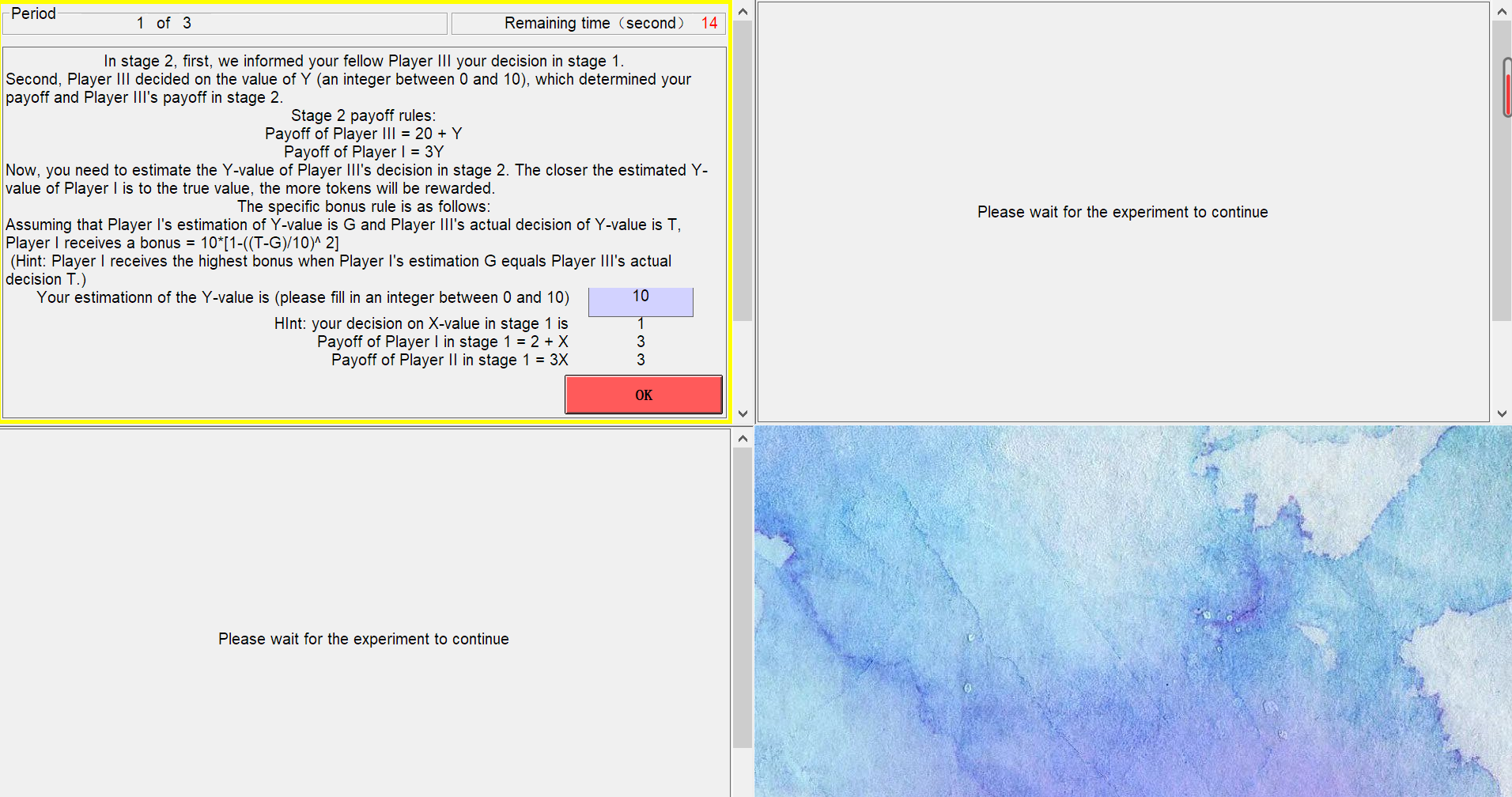


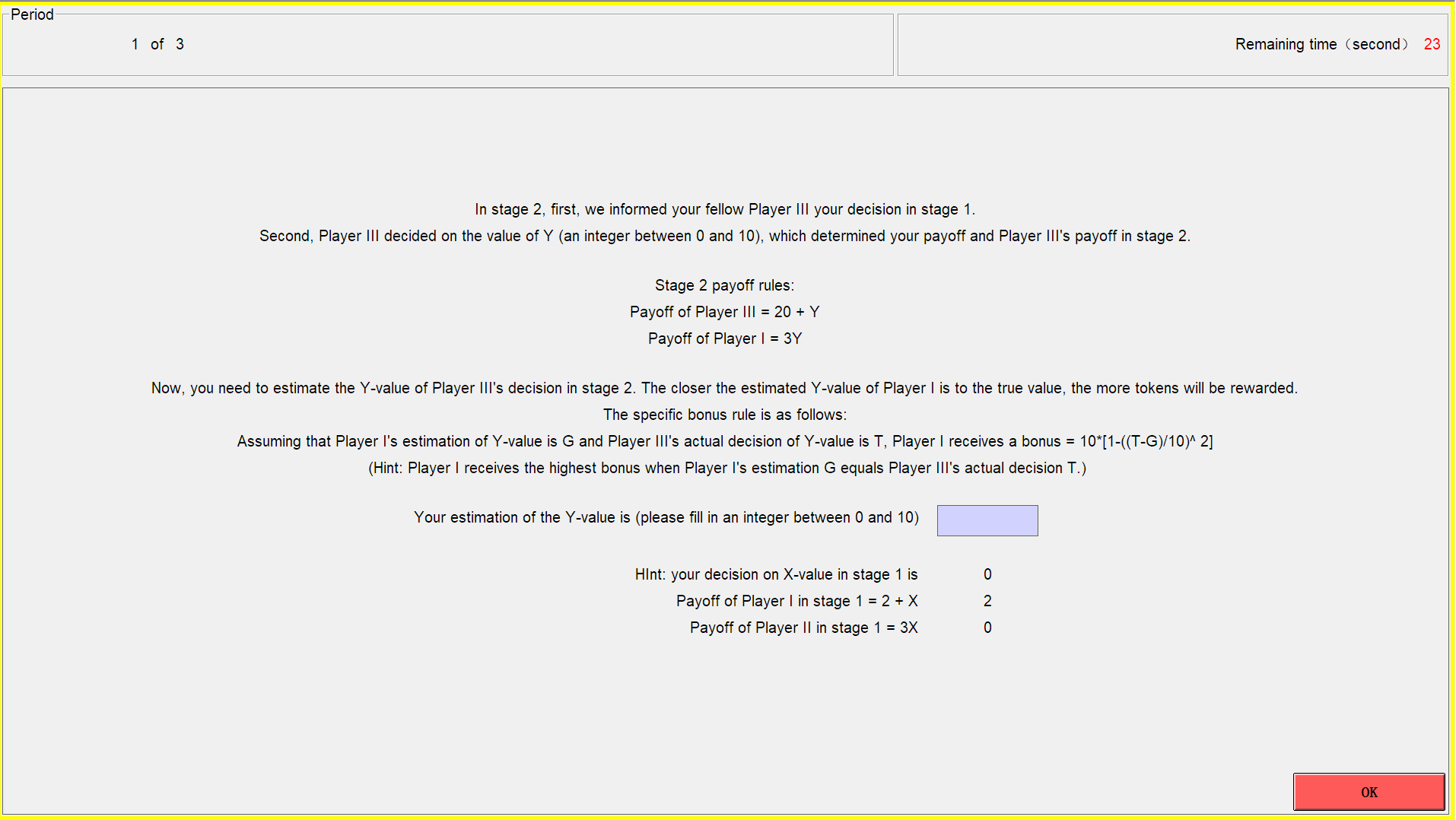


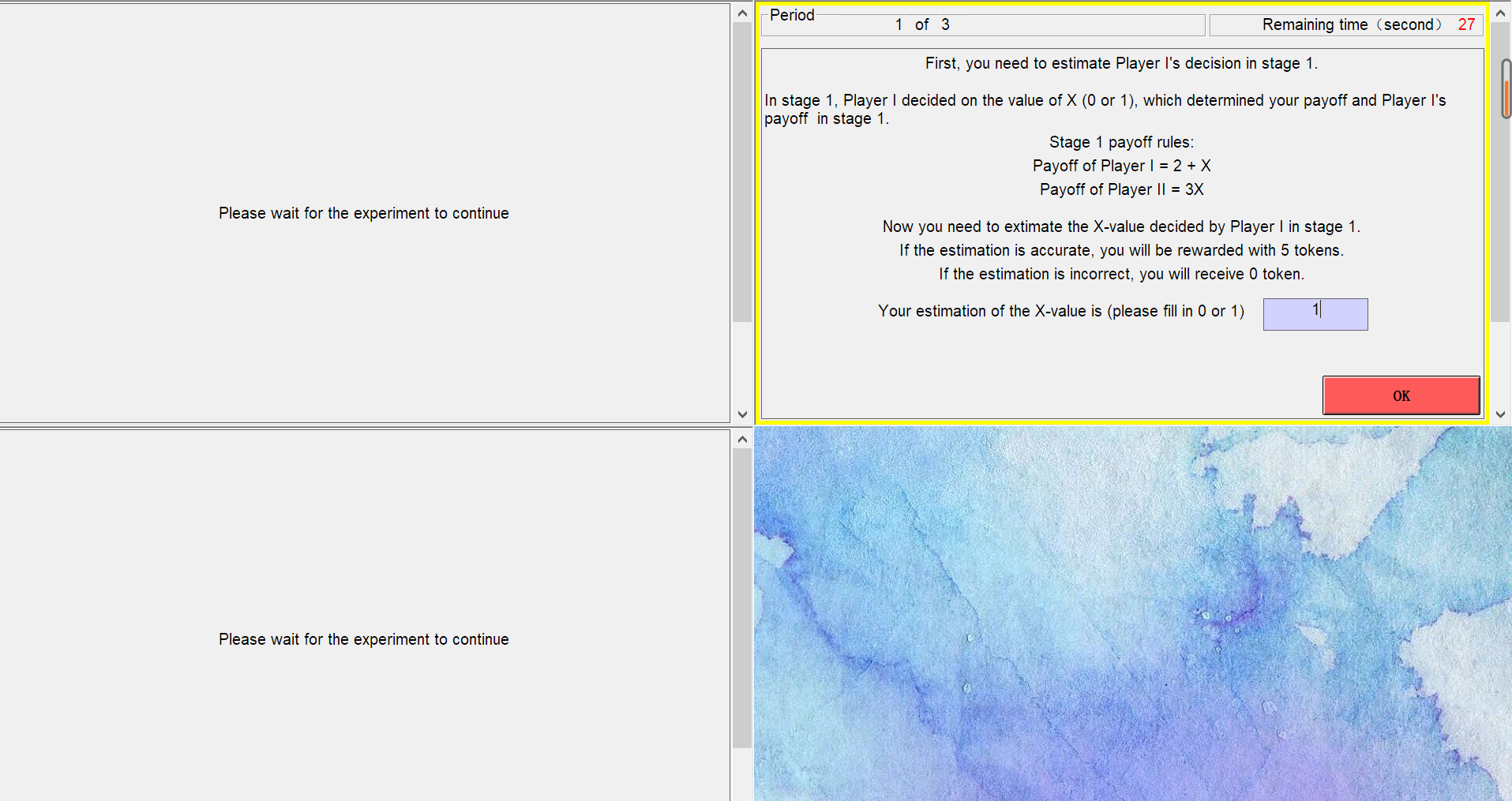

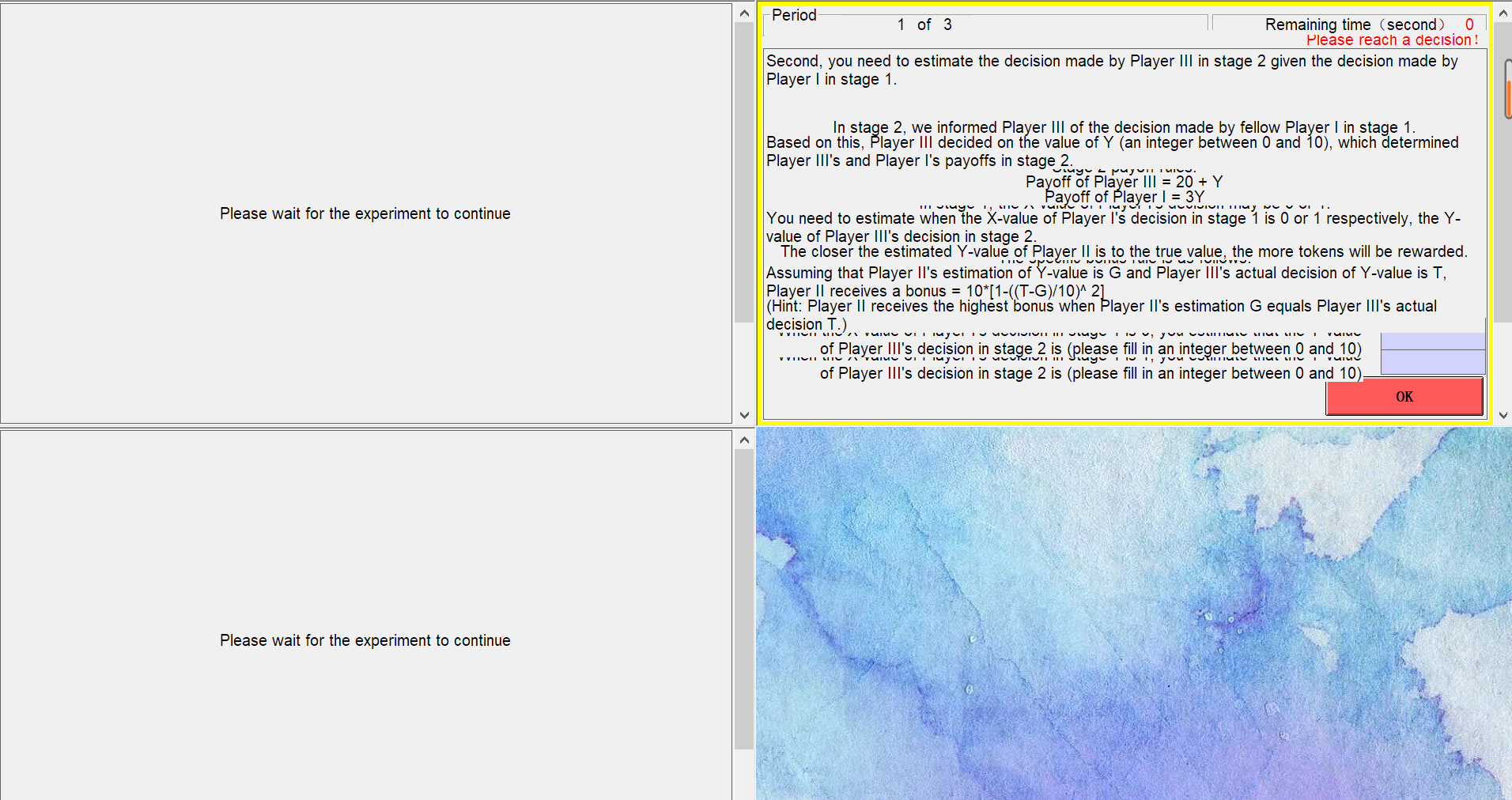


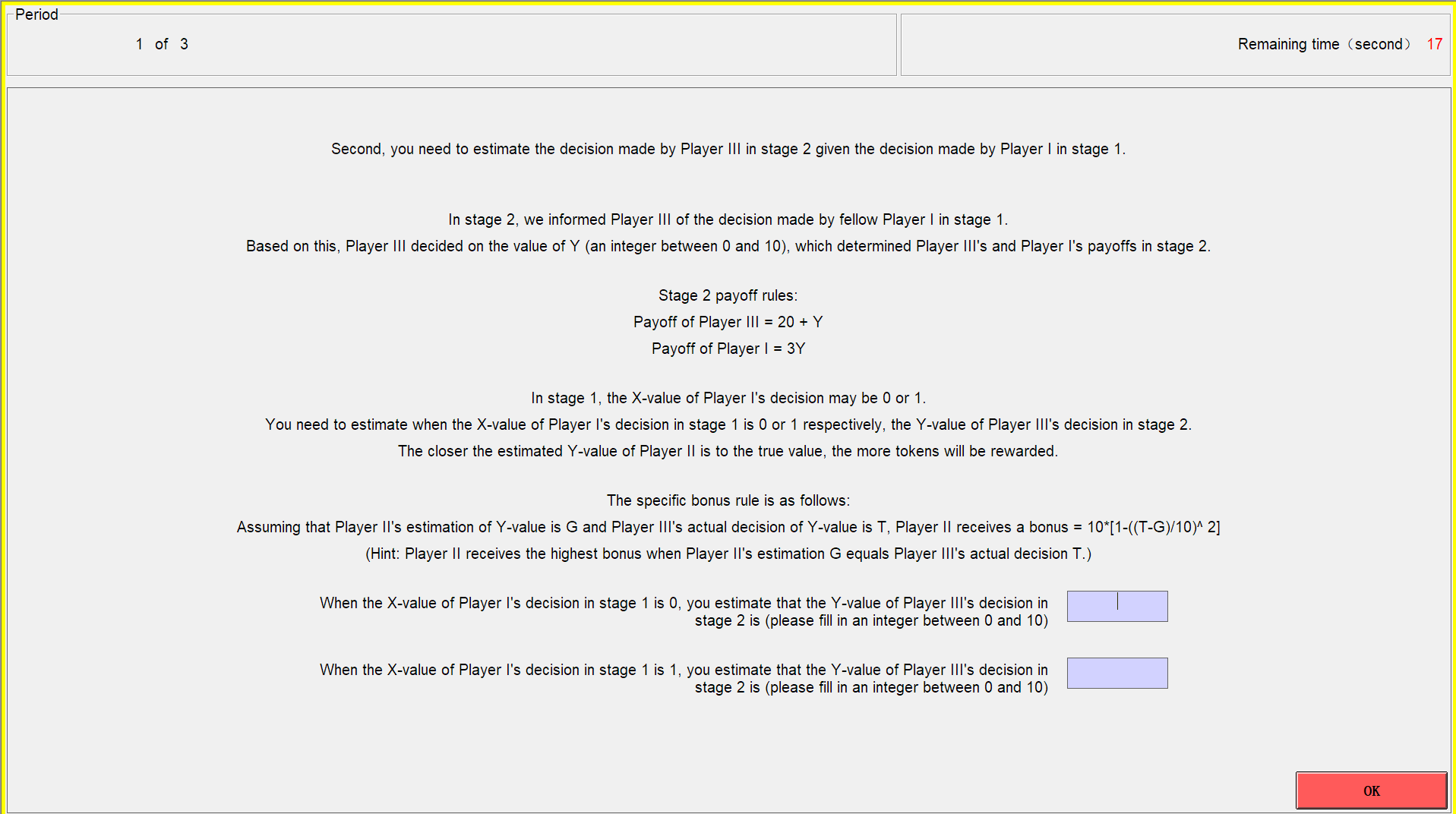


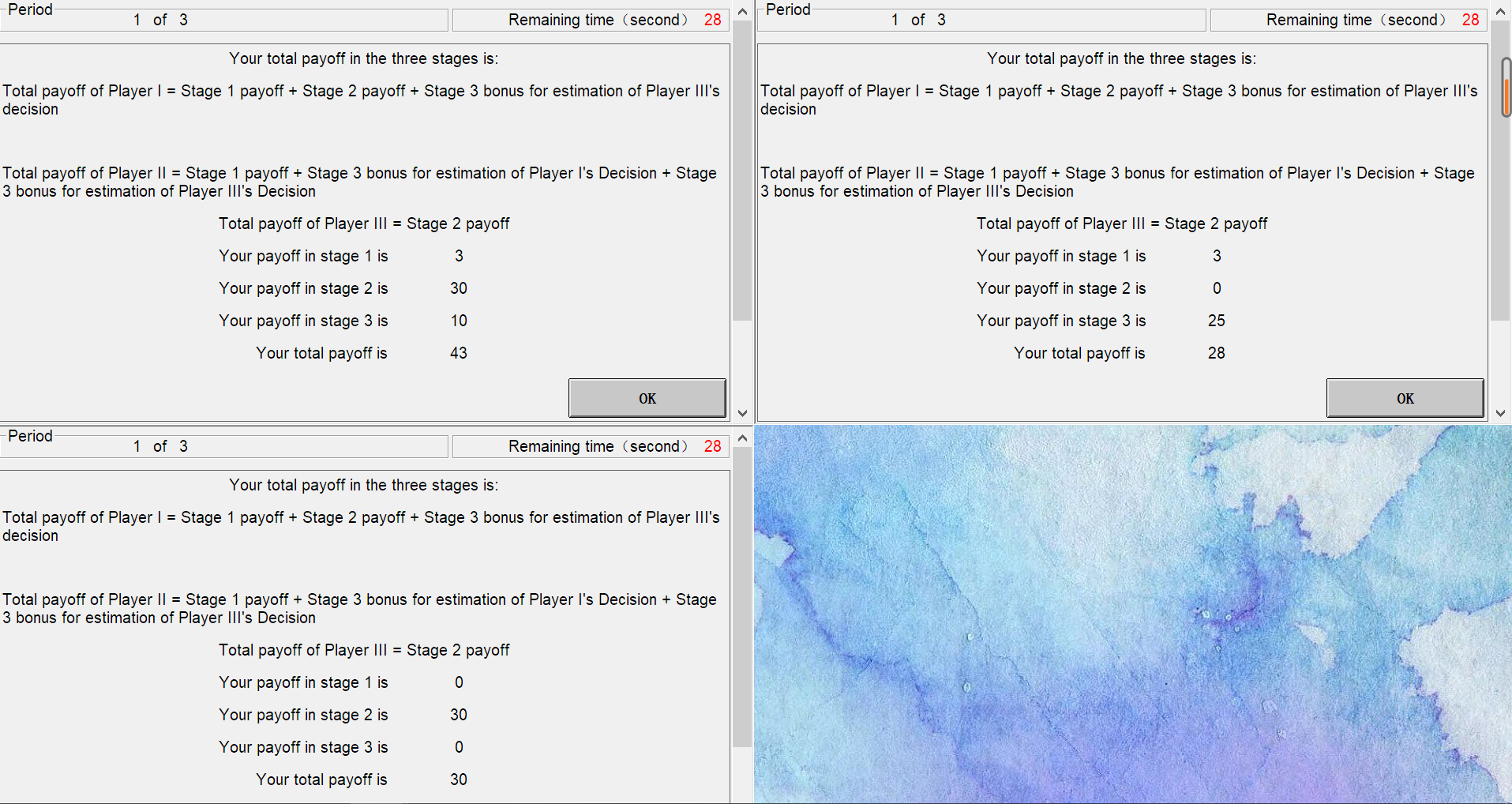


After three rounds:


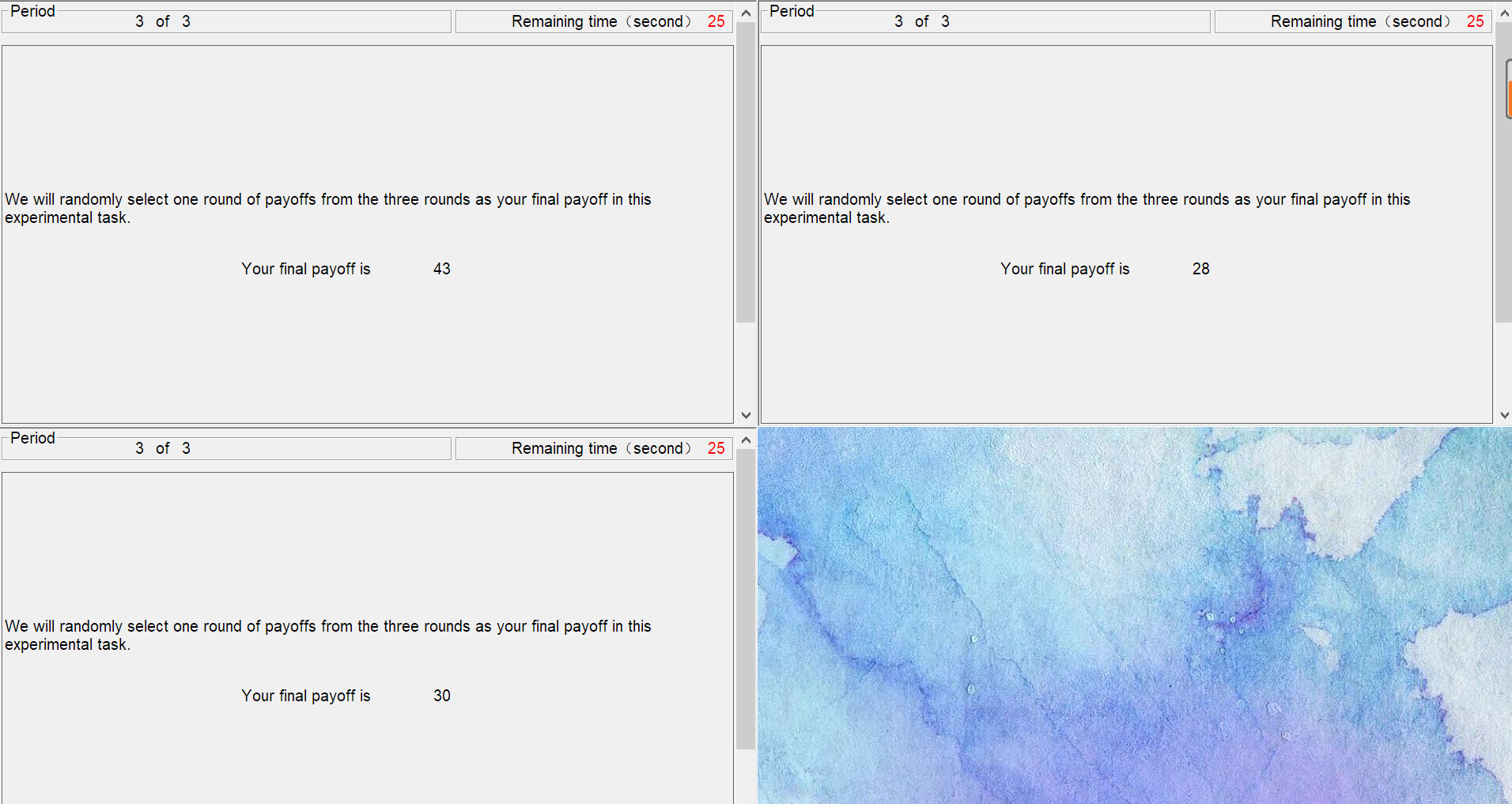

Supplement: Supplementary file 1 — Supplementary Information. [file 41598_2023_41256_MOESM1_ESM.docx]
